# Actin Waves Do Not Boost Neurite Outgrowth in the Early Stages of Neuron Maturation

**DOI:** 10.3389/fncel.2017.00402

**Published:** 2017-12-18

**Authors:** Simone Mortal, Federico Iseppon, Andrea Perissinotto, Elisa D'Este, Dan Cojoc, Luisa M. R. Napolitano, Vincent Torre

**Affiliations:** ^1^Neurobiology Department, International School for Advanced Studies, Trieste, Italy; ^2^Department of NanoBiophotonics, Max Planck Institute for Biophysical Chemistry, Göttingen, Germany; ^3^Optical Manipulation Lab, Istituto Officina dei Materiali (CNR), Trieste, Italy

**Keywords:** actin waves (AWs), growth cones (GCs), Myosin IIB, β-cyclodextrin, RhoGTPases

## Abstract

During neurite development, Actin Waves (AWs) emerge at the neurite base and move up to its tip, causing a transient retraction of the Growth Cone (GC). Many studies have shown that AWs are linked to outbursts of neurite growth and, therefore, contribute to the fast elongation of the nascent axon. Using long term live cell-imaging, we show that AWs do not boost neurite outgrowth and that neurites without AWs can elongate for several hundred microns. Inhibition of Myosin II abolishes the transient GC retraction and strongly modifies the AWs morphology. Super-resolution nanoscopy shows that Myosin IIB shapes the growth cone-like AWs structure and is differently distributed in AWs and GCs. Interestingly, depletion of membrane cholesterol and inhibition of Rho GTPases decrease AWs frequency and velocity. Our results indicate that Myosin IIB, membrane tension, and small Rho GTPases are important players in the regulation of the AW dynamics. Finally, we suggest a role for AWs in maintaining the GCs active during environmental exploration.

## Introduction

The actin cytoskeleton is a highly dynamical system that facilitates the transduction of mechanical signals and generates the intracellular forces required for many cellular functions such as cell motility, polarization, active cell shape control, neurite outgrowth, and exocytosis (Madden and Snyder, 1998; Morales et al., 2000; Pollard and Borisy, 2003). Growth Cones (GCs) are located at the tip of neurites and are composed of a veil-like structure, referred to as the lamellipodium, from which thinner filopodia emerge (Lowery and Van Vactor, 2009; Dent et al., 2011). A characteristic feature of all these processes is the dynamical assembly of filamentous actin (F-actin) from its subunits (G-actin) that sustains a constant and rapid reshaping of the actin network. Actin Waves (AWs) are growth cone–like structures that emerge at the base of neurites, slowly migrating up to their tips with a speed of ~2–3 μm/min, flaring the plasma membrane during transit (Ruthel and Banker, 1998, 1999; Flynn et al., 2009; Katsuno et al., 2015). AWs have been observed mainly in growing neurons and are present in cultured primary hippocampal neurons as well as in organotypic slices (Ruthel and Banker, 1998; Flynn et al., 2009; Katsuno et al., 2015). They have been proposed to be associated with outbursts of neurite growth and to constitute, in concert with microtubules, a transport mechanism that brings actin and actin associated proteins toward the GC (Flynn et al., 2009; Katsuno et al., 2015; Winans et al., 2016).

An important component of the actin dynamics is thought to be the acto-myosin force-generating machinery. Myosin II, a central player during cell contraction, is an actin-dependent molecular motor moving toward the plus (barbed) end of the actin filament. Myosin IIA and IIB are the most prominent type II myosin expressed in neurons migrating within the CNS (Golomb et al., 2004). In particular, Myosin IIB is required for maintaining normal growth cone shape and traction force, polarization size, and actin organization (Bridgman et al., 2001). Although the role of Myosin II in the rear of motile cells is conventionally associated with mechanical force generation and contraction, it is now clear that Myosin IIB also has a specific role in driving actin network disassembly (Wilson et al., 2010). Over the last years it has also become clear that Myosin II is regulated by the Rho family members of small GTPases (Rac, RhoA, and Cdc42). In general, RhoA/Rho-kinase (ROCK) activates Myosin II contractility, whereas its effector PAK often regulates Myosin II negatively decreasing cell contractility (Lee et al., 2010). The Rho GTPases act as molecular switches to control signal transduction pathways by cycling between a GDP-bound, inactive form, and a GTP-bound, active form (Raftopoulou and Hall, 2004). They have also been considered as putative Nucleation Promoting Factors (NPFs) (Weiner et al., 2007; Holmes et al., 2012) that modulate actin filament nucleation influencing many aspects of the cell behavior such as cell migration. Indeed, Cdc42 induces actin polymerization to generate filopodia often seen at the front of migrating cells, while Rac has a key role in generating a protrusive force through the localized polymerization of actin (Nobes and Hall, 1995; Raftopoulou and Hall, 2004; Ridley, 2011). Consistent with this view, it is not surprising that the use of a Raichu FRET reporter (Komatsu et al., 2011) has revealed an increase of Rac activity in and behind the AWs showing a role for Rac in generating the AWs (Winans et al., 2016). In addition, Machacek et al (Machacek et al., 2009) showed a tight correlation between GTPases activation and morphological edge dynamics and in (Wu et al., 2013), waves of Cdc42 proved to be correlated in both space and time with waves of F-actin.

We investigated the function of AWs and their regulating mechanisms using live cell-imaging. Our work aimed to solve three main issues: (1) Do AWs really promote neurite outgrowth? (2) Which is the AWs subcellular organization and to what extent AWs and GCs are similar? (3) What is the mechanism allowing the migration of AWs along the neurites? Indeed, AWs might create tension and exert pulling forces along the neurites (Ruthel and Banker, 1999; Lamoureux et al., 2002; Tomba et al., 2017). In the present manuscript, we use STED nanoscopy and live-cell imaging to tackle these questions.

Our results show that AWs do not contribute to neurite outgrowth regardless of the substrates used (matrigel/laminin, poly-D-lysine, poly-L-ornithine). We also observed that, in accordance with Flynn et al. (2009), as an AW approaches the distal neurite, the GC moves closer to the incoming AW and merges with it. Furthermore, our data highlight that, despite the similarities in shape and appearance, AWs and GCs exhibit a different response to the blebbistatin, a cell permeant inhibitor of Myosin II (Kovács et al., 2004), that leads to a substantial collapse of the GC but not of the AW. We then investigated the cross-talk between AWs and Rho GTPases and our data support the idea that cytoskeletal dynamics and Rac1/Cdc42 activities are tightly coupled at subcellular levels.

## Materials and methods

### DNA constructs

mCherry-Lifeact-7 was a gift from Michael Davidson (Addgene plasmid #54491) and was verified by full-length sequencing.

### Primary hippocampal neuron culture and transfection

Hippocampal neurons from Wistar rats (P2-P3) were prepared in accordance with the guidelines of the Italian Animal Welfare Act, and their use was approved by the Local Veterinary Service, the SISSA Ethics Committee board and the National Ministry of Health (Permit Number: 2848-III/15) in accordance with the European Union guidelines for animal care (d.l. 26, March 4th 2014 related to 2010/63/UE and d.1. 116/92; 86/609/C.E.). The animals were anesthetized with CO_2_ and sacrificed by decapitation, and all efforts were made to minimize suffering. The coverslips were coated with 50 μg/ml poly-L-ornithine (Sigma-Aldrich, St. Louis, MO, USA) overnight and with Matrigel before cells seeding (Corning, Tewksbury MA, USA). In control experiments, the coverslips were coated with 50 μg/ml poly-L-ornithine (Sigma-Aldrich, St. Louis, MO, USA) overnight or 0.5 mg/ml poly-D-lysine (Sigma-Aldrich, St. Louis, MO, USA) for 1 h at 37°C. Dissociated cells were plated at a concentration of 4 × 10^4^ cells/ml in Minimum Essential Medium (MEM) with GlutaMAX™ supplemented with 10% Fetal Bovine Serum (FBS, all from Invitrogen, Life Technologies, Gaithersburg, MD, USA), 0.6% D-glucose, 15 mM Hepes, 0.1 mg/ml apo-transferrin, 30 μg/ml insulin, 0.1 μg/ml D-biotin, 1 μM vitamin B12 (all from Sigma-Aldrich), and 2.5 μg/ml gentamycin (Invitrogen). The neuronal cultures were maintained in an incubator at 37°C, 5% CO_2_, and 95% humidity. Hippocampal neurons were transfected immediately or 24 h after dissection with the LifeAct plasmid using Lipofectamine 3000® reagent (Invitrogen) following the manufacturer's protocol, and imaged 1 day after transfection. To stain the membrane, cells were incubated with Vybrant DiI (5 μL/mL) (Thermofisher) for 20 min at 37°C. SiR-Actin (Spirochrome) was used at 200 μM, incubating it for 30 min at 37°C.

### Drug application

Blebbistatin (Sigma) and β-cyclodextrin (Sigma) were used at a final concentration of 20 and 250 μM respectively. ML141 (TOCRIS bioscience, Bristol, UK) was used at a final concentration of 10 μM (for low concentration experiments) and 30 μM (for high concentration experiments); EHT1864 (TOCRIS) was used at a final concentration of 10 μM. All inhibitors used were added after about 60–90 min of imaging, and the acquisition continued for at least 90 min after addition, at the conditions described in paragraph Live Cell Imaging.

### STED nanoscopy

Hippocampal neurons prepared from P0-P2 Wistar rats were plated on Poly-ornithine/Laminin coated coverslips. At DIV 1, cells were washed with PBS and fixed in 4% PFA and 0.25% Glutaraldehyde in PHEM buffer (60 mM PIPES, 25 mM HEPES, 10 mM EGTA, 2 mM MgCl_2_, pH 6.9) for 20 min at room temperature, quenched with ammonium chloride and glycine (100 mM each) for 5 min, permeabilized with 0.1% Triton X-100 for another 5 min and blocked in PBS supplemented with 1% BSA for 30 min. Primary antibodies against β-III Tubulin (Abcam, cat. ab7759), Myosin-IIB (Sigma, cat. M 7939) and Actin (Phalloidin) were incubated in PBS for 1 h or overnight at 4°C. Secondary antibodies (sheep anti-mouse, Dianova, cat. 515-005-003; goat-anti-rabbit, Dianova, cat. 111-005-003) were labeled with STAR580 (Abberior, cat. 1-0101-005-2), and Alexa 488 secondary antibodies (Invitrogen, goat anti-mouse IgG, cat. A11001; goat anti-rabbit, cat. A-11008) were used for confocal imaging. Phalloidin was coupled to STAR635 (Abberior, cat. 2-0205-002-5). Both secondary antibody and phalloidin incubations were performed in PBS for 1 h at room temperature or overnight at 4°C. Samples were then mounted in Mowiol supplemented with DABCO. Imaging was performed on a two-color Abberior STED 775 QUAD scanning microscope (Abberior Instruments GmbH, Göttingen, Germany) equipped with 488 nm, 561 nm, and 640 nm pulsed excitation lasers, a pulsed 775 nm STED laser, and a 100x oil immersion objective lens (NA 1.4).

### Live cell imaging

Live cell imaging experiments were performed on an epi-fluorescence microscope (Olympus IX-83, Olympus) equipped with LED illumination (λ = 590 nm for LifeAct-mCherry; λ = 530 nm for Vybrant DiI; λ = 660 nm for SirActin, all purchased from Thorlabs). During all imaging experiments cells were kept at 37°C, 5.0% CO_2_, 95% humidity by using an imaging chamber incubator (Okolab, Pozzuoli, Italy). Time-lapse images were taken for up to 10 h, with 1 s exposure time, every 1–5 min using a 20X air objective (Olympus, NA = 0.75) or every 5-10s using a 40X oil immersion objective (Olympus, NA = 1.3). All acquisitions were done with a CCD sensor at 12bit depth (ORCA-D2, Hamamatsu).

### Image analysis tools

Image analysis was performed using ImageJ software (NIH) and a custom-build MATLAB code (Mathworks). The software consists in three main modules: the first image of the sequence is processed with a user customizable 2d Gaussian filter in order to remove the noise. The user then needs to select a point inside the soma of the cell under investigation. This action identifies the origin of the coordinate system that allows the automatic extraction of the cell body. Subsequently, a phase congruency algorithm is applied in order to detect line features such as neurites: this results in a black and white map where the user selects the position of the neurite hillock and its edge (Kovesi, 2000; Wu et al., 2010). The identification of the soma and neurite geometries allows the user to restrict the area of the possible locations in which the neurite hillock and the neurite edge can be found. In our experiments, the length of the neurite was evaluated as the minimum path necessary to connect the two points passing through the above-threshold pixels, defining the neurite in the map. The AW position was also selected manually by the user and, applying the method above mentioned, the distance covered along the neurite was evaluated. Finally, the algorithm was applied to all the images in the sequence using the same parameter, adjusting the position of the soma and the three points on the neurite (the neurite hillock, its edge and AW position) according to the corresponding map obtained from the phase congruency algorithm. The user supervised the whole operation, correcting the algorithm when necessary.

### Morphological analysis of actin waves and growth cones

For the morphological analysis of each AW, the area was determined manually at different time-points of the wave progression along the neurite, before and after drug addiction. The mean AW area throughout the neurite was then obtained by averaging the areas previously studied. For the morphological analysis of the GCs, the area was determined manually at different time-points of the wave progression and also every 30 s for about 5–10 min after arrival and merging of the AW with the growth cone. The mean GC areas were then obtained by averaging the areas previously calculated.

### Intensity line scans

Line scans were performed with ImageJ software. Images were background subtracted and thresholded to select the neurite of interest, and then a line was traced in the neurite to have an intensity profile trace. Traces were then aligned to the half-maximum value of the front of the actin wave (oriented toward the growth cone). Half maximum values were determined by manual identification of the maximum actin signal at the front half of the actin curve. Traces were then computationally aligned using a custom-built MATLAB software.

### Myosin puncta density measurements

STED images were background subtracted and thresholded to highlight Myosin puncta. Regions of Interest were traced in the central and peripheral regions of AWs and GCs, in the rear and front halves of the AWs, and in two neurite sections of the same area for each AW (8–12 μm^2^) immediately before and after the AWs. The areas were then analyzed through the SynPAnal software (Danielson and Lee, 2014) to detect the puncta density in the areas of interest. In the same regions, the actin mean intensity was calculated using an ImageJ software, and the resulting puncta density measurements were normalized for the highest actin intensity data for each AW and GC that have been considered.

### Analysis of the actin wave position, velocity, and frequency along the neurite

All the analyses of the AW position were performed using an ImageJ program and custom-built codes written in MATLAB (Mathworks) (see Image Analysis Tools paragraph). Time-lapse sequences were background corrected and thresholded to select the neurite of interest. The positions of the soma and of the neurite's end were tracked automatically, whereas the AW position along the neurite was tracked manually. The distances calculated from the soma were then extrapolated and plotted as a function of time. The time-points when no wave was traveling along the neurite were eliminated. The velocity of the AW was calculated by extracting the distance covered and the time taken from the position data previously described, then by dividing distance over time. The frequency of AW was calculated as AW per hour by manually counting all the AW events before and after drug addiction.

### Statistical analysis

All results have been obtained from at least three independent experiments and expressed as the mean ± S.E.M. Experimental data were analyzed with Student's *t*-test or U-Mann Whitney test using a SigmaPlot 10.0 software (Systat Software Inc.). Differences among samples were considered statistically significant when *p* < 0.05.

## Results

**Figure 1:**
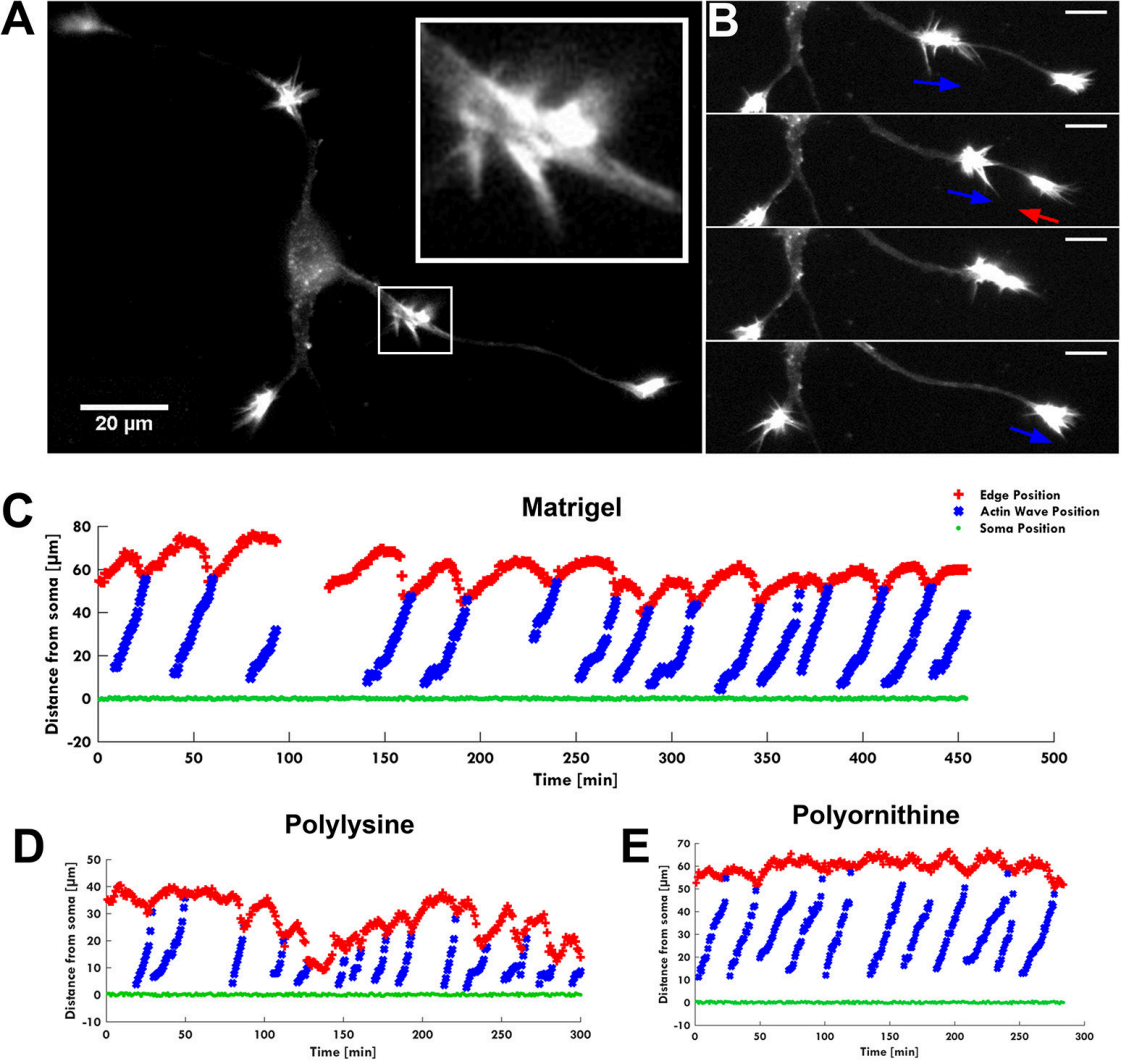
The AW pulling effect on GC. **(A)** Fluorescence image of F-actin showing an actin wave advancing along a neurite with its characteristic growth cone-like shape (white square). The insert shows a higher magnification of the growth cone-like AW. **(B)** Time-lapse images of the neurite shown in **(A)**. When an AW approaches the distal neurite, the GC retracts several microns to reach the wave, then merges with it and advances following the wave arrival. Blue arrows indicate AW direction; red arrows indicate GC retraction. Scale bar refers to **(A,B)** = 20 μm. **(C–E)** Plots showing the progression of several AWs (blue) shown in **(A,B)** and Supplementary Video 1 from the soma (green) along the neurite. The origin of the axes is set to the initial soma position. The edge of the neurite (red) shows the retraction/growth cycles of the GC upon arrival of the AW. The mean velocity of AWs is 2.2 ± 0.4 μm/min. For these experiments, the hippocampal neurons were plated on different substrates: Matrigel/Laminin **(C)**, poly-D-lysine **(D)** and poly-L-ornithine **(E)**.

Due to their highly dynamic behavior, study of the AWs requires live imaging. Hence, we performed long-term live cell imaging of AWs on rat hippocampal cells plated on Matrigel/Laminin at 1–2 days *in vitro* (1–2 DIV) using the actin label mCherry-LifeAct (Riedl et al., 2008; Winans et al., 2016). In this experimental setting, the morphology and dynamics of the AWs were comparable to what had been previously reported (Ruthel and Banker, 1998; Flynn et al., 2009; Katsuno et al., 2015; Winans et al., 2016). Indeed, hippocampal AWs exhibited a growth cone-like morphology and behavior forming filopodia and lamellipodia on one or both sides of the neurite shaft and travel mainly in an anterograde fashion in our 1 and 2 DIV imaging windows (Figure 1A; Supplementary Video 1). The analysis of 114 neurites showed that the AWs are generated at a median frequency of 2–3 per hour and move at an average speed of 2–3 μm/min, in agreement with previous reports. Strikingly, no difference in AWs' behavior was observed plating the hippocampal neurons on different substrates such as poly-D-lysine and poly-L-ornithine (Figure 1; Supplementary Videos 2, 3).

LifeAct is known to induce bundling of actin filaments (Riedl et al., 2008). Therefore, to rule out any effect of our experimental setup on the dynamics of the AWs, we tested other staining approaches. As an alternative actin label, we used SiR-Actin (Lukinavicius et al., 2014), a membrane-permeable based on the jasplakinolide, a drug that promotes actin polymerization by enhancing the rate of filament nucleation (Bubb et al., 1994). SiR-Actin caused a decrease of the AWs number and of actin motion, presumably due to the residual activity of jasplakinolide (Supplementary Figure 1) that was shown to freeze AWs (Winans et al., 2016). Therefore, we tested the membrane marker Vybrant DiI which is well known to not interfere with actin dynamics. Vybrant DiI enabled the visualization of AWs (Supplementary Video 4), which exhibited velocities (2–3 μm/min) and frequencies (2–3 AW/h) similar to what observed when mCherry-LifeAct is present. Hence, we concluded that LifeAct does not have any effect on the AWs dynamics and can be used in this experimental system.

### Actin waves do not boost neurite elongation

**Figure 2:**
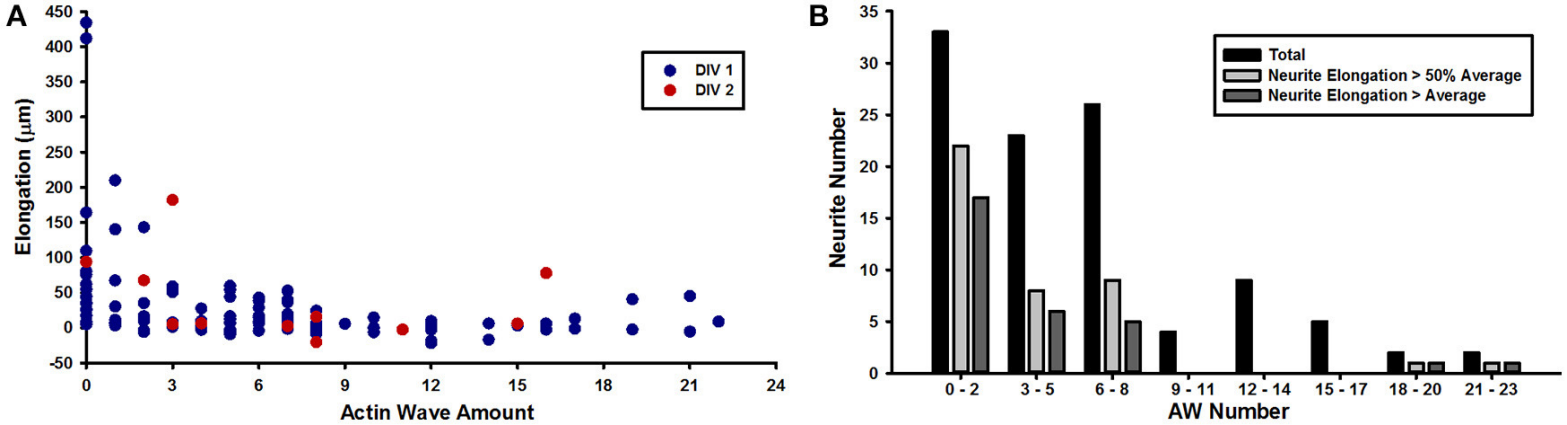
AWs inhibit neurite elongation**. (A)** Graph showing the elongation of the neurites (y axis) per number of actin waves (x axis) imaged at 1–5 min intervals for 8 h. Red dots refer to DIV2 hippocampal culture; blue dots refer to DIV1 hippocampal culture. *n* = 126 neurites **(B)** Quantification of the number of neurites (y axis) per number of actin waves (x axis). *n* = 126 neurites. Black bars refer to all DIV1-DIV2 neurites that present 0–2, 3–5, 6–8, 9–11, 12–14, 15–17, 18–20, or 21–23 AWs; light gray bars refer to neurites that elongate more than 50% of the average length; dark gray bars refer to neurites that elongate up to the average length.

The live cell imaging of the rat hippocampal cells showed that as an AW moves toward a distal GC at the tip of a neurite, the GC retracts by about 15–20 μm approaching the incoming AW and merges with it (Figure 1B). This pulling effect, already described by Flynn et al. (2009), was observed in almost all neurites both 1 and 2 DIV. The arrival of AWs is associated to a transient retraction of the GC and also to a transient increase of the GC size (Supplementary Figure 2) so that the cyclic behavior is observed. These events, however, do not result in a net neurite elongation (Figure 1C). We then analyzed in detail the relation between AWs reaching the neurite tips and neurite outgrowth (Figure 2A) and we could not observe any positive correlation between the number of migrating AWs and neurite elongation in a total of 126 neurites emerging from the cell body of hippocampal neurons (Figure 2A and Supplementary Figure 3). Our data clearly show that neurites exhibiting either no or a small number of AWs grow much more than neurites with a larger quantity of AWs (Figures 2A,B): the former can grow up to 400–450 μm, while the latter < 50 μm or do not grow at all. In contrast with what reported in previous papers (Flynn et al., 2009; Winans et al., 2016), no difference was observed between 1 and 2 DIV (Figure 2A).

**Figure 3:**
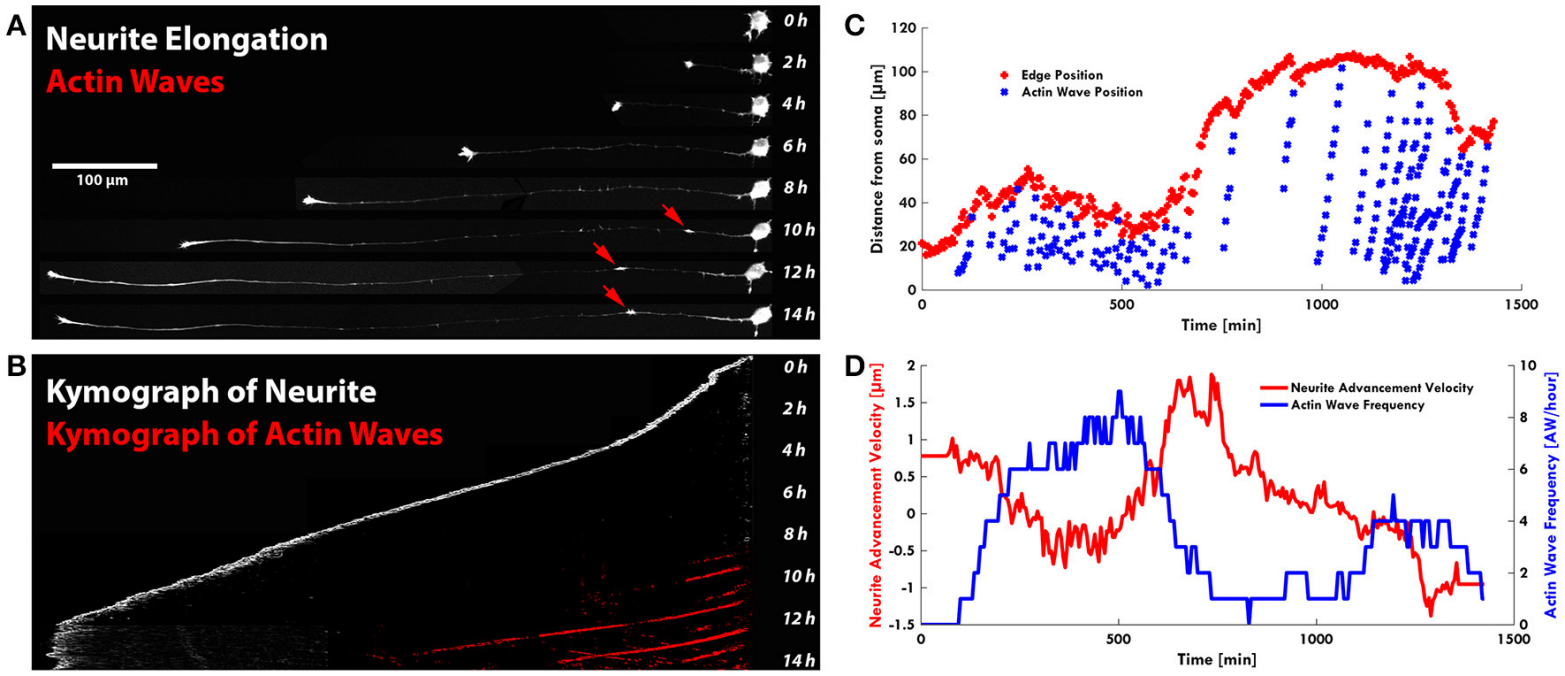
AWs are not associated to neurite outgrowth. **(A)** Timelapse images of the neurite shown in Supplementary Video 5: the neurite grows up to 450 μm in the presence of a few AWs (red arrows) (one of the two points in Figure 2A with no AWs). Scale bar = 100 μm **(B)** Kymograph generated from the time lapse images of the F-actin expressing neurite shown in **(A)**. **(C)** Plot highlighting the progression of the AWs (light blue cruises) along the neurite shown in Supplementary Figure 4. The edge trace (red cruises) highlights that neurite outgrowth occurs with a few AWs, while in the presence of high frequency of AWs, in red, the neurite does not grow. **(D)** Graph highlighting in red the advancement velocity (μm/min) of the neurite shown in **(C)** and in blue the AWs frequency in function of time.

**Figure 4:**
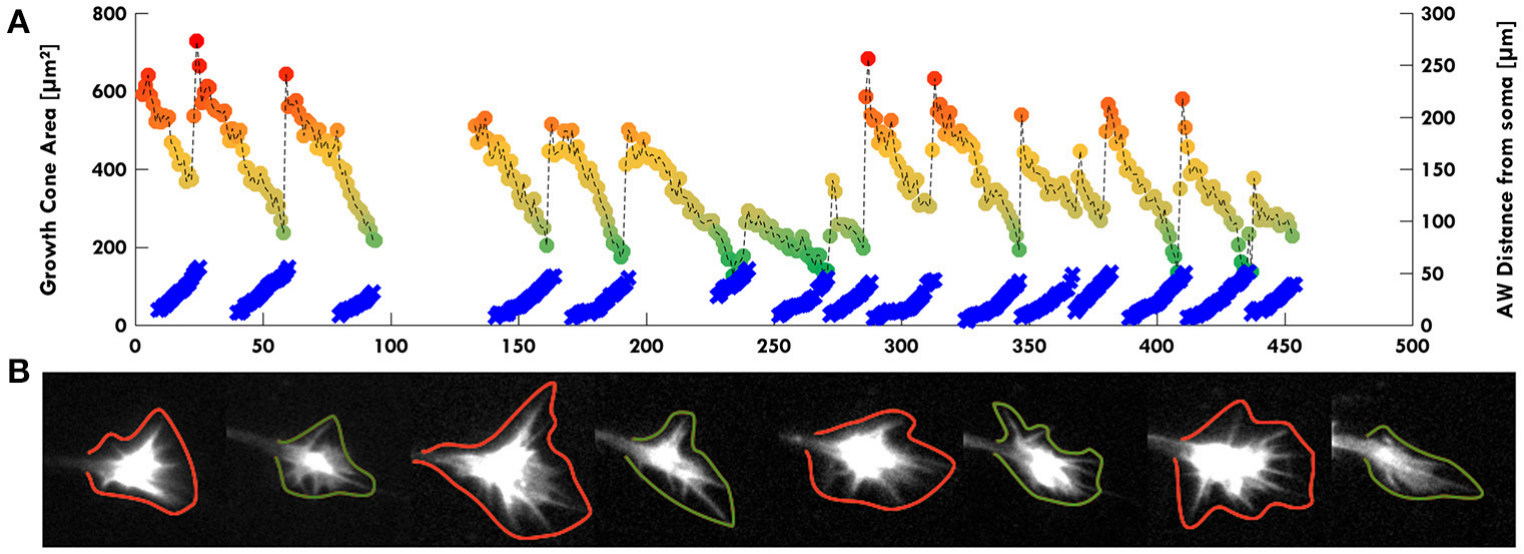
AWs increase GC volume. **(A)** Graph highlighting in color scale (from red to green dots) the change of the GC Area (μm^2^ on left scale) and in light blue the AW distance from the cell soma (μm on right scale) in function of time (minutes). The GC size is maximum (red dots) when the AW arrives to the tip of the neurite and merges with the GC itself. The GC minimum size (green dots) is reached immediately before the arrival of the AW when the GC is pulled backwards, and the AW is close to the GC without merging. The dark yellow dots indicate the intermediate states from the maximum GC size (red dots) to the minimum one (green dots). All the points corresponding to the GC size are connected with a dashed line. **(B)** Examples of some timelapse images used in **(A)**. GCs with green contours refer to green dots in **(A)**; GCs with red contours refer to red dots in **(A)**. We observed that AWs induce an increase of the GC volume with filopodia sprouting (see also Supplementary Figure 2) and this suggests a major role for AWs in regulating GC sensing the surrounding environment.

Remarkably, in several developing neurons, we observed a continuous neurite growth with an almost constant elongation speed in the absence of migrating AWs (2–3 μm/min), which slowed down (0.5 μm/min) in the presence of AWs (Figure 3A). The kymographs of the neurite tip (white) and AWs (red) in Figure 3B show that when AWs started to reach the GC tip its elongation stopped completely. When developing hippocampal DIV1 or DIV2 neurons were stained with Vybrant DiI we could follow for up to 20 h—and even for 1 day—the neurite growth and the appearance and migration of AWs. In the example shown in Figure 3C, during the initial 10 h of observation many AWs were seen propagating along the neurite shaft and reaching the tip (red cross) but the neurite (blue cross) did not grow and no significant neurite elongation was observed (Figure 3C). In the same experiment, when the frequency of AWs decreased substantially the neurite started to grow and in the following 6 h only two AWs were observed. Finally, AWs appeared again, the neurite growth stopped and a small retraction was observed (Figure 3C). Within the same time window (Figure 3D) we computed the velocity v of the neurite tip (blue line) and the frequency of migrating AWs (red line) and we found that when the frequency increased, the velocity decreased concomitantly and vice versa, suggesting that there is a negative correlation between neurite advancement and frequency of appearance of AWs. We obtained another five live cell experiments with a duration longer than 6 h and in all these experiments we observed a negative correlation between AWs frequency and neurite elongation. In order to have a more complete picture of the AWs' role, we computed the GC area (color scale line) and the AW distance from the soma (blue line) from the experiment reported in Figure 1. Interestingly, the GC lost between 30 and 70% of its original area in 30–60 min after fusion with the wave, in the absence of any significant elongation (Figure 4A). When we computed the mean area of the GC before and in the 5–10 min after the AW arrival, we observed a two-fold increase in the GC area (Supplementary Figure 2), confirming previous findings on GC size and dynamicity change after the AW arrival (Flynn et al., 2009). These results indicate that AWs have a paramount role in maintaining highly motile the GC which can explore the surrounding environment (Figure 4B).

### Myosin IIB is required for AW formation and structure

**Figure 5:**
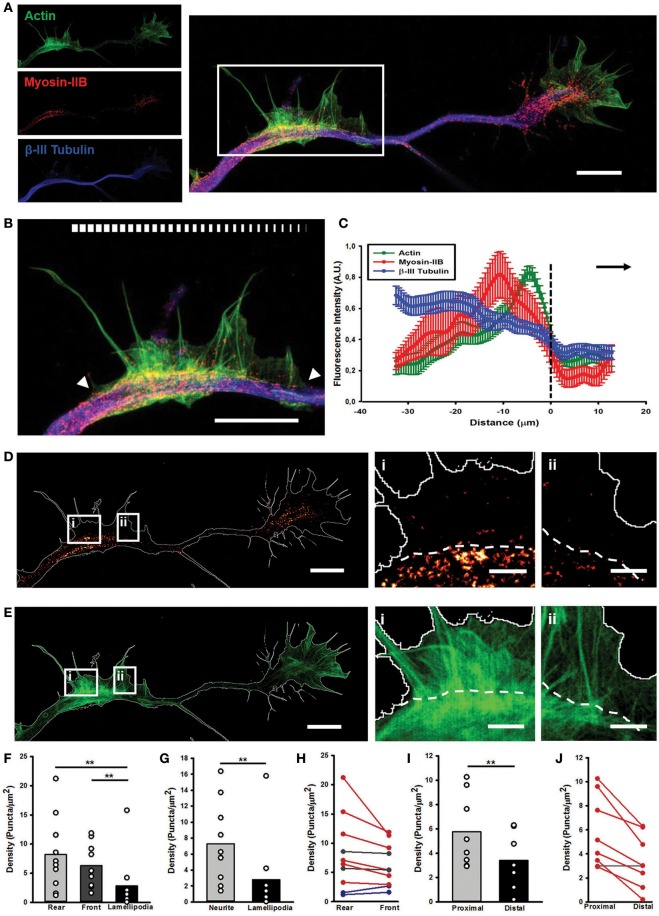
STED microscopy reveals Myosin IIB in AWs. **(A)** STED image of a rat hippocampal neuron at 1 DIV stained with actin (phalloidin, green), non-muscle myosin IIB (red) and β-III tubulin (blue) shows the presence of myosin IIB in the GC and AW. The white box highlights the AW. The insets on the left are the single channel images. Scalebar: 5 μm **(B)** Magnification of the AW [white box in **(A)**] highlighting that myosin IIB is brightest in the rear region of the AW. The white arrowheads indicate the beginning and the end of the AW, and the white bar mimics the myosin intensity. Scalebar = 5 μm **(C)** Averaged line scans (see Methods) show that myosin IIB is highly concentrated at the rear of advancing actin wave. Dashed line indicates AW front, and black arrow highlights wave direction. *N* = 9 neurites. Measurements are performed on STED images. All traces were normalized by mean intensity and smoothed before averaging (Winans et al., 2016). Data shown as Mean ± SEM. **(D,E)** STED images of the myosin IIB **(D)** and actin **(E)** channels showing that the myosin brightest puncta concentrate in the GC and in and behind the AW. The panels on the right show two magnifications of sections from the back (i) and front (ii) regions of the AW, with a clear border between the neurite and the emerging peripheral lamellipodia, highlighted by the white dashed lines. Scalebars in **(D,E)** = 5 μm. Scalebars in (i, ii) = 1 μm. **(F)** Histogram showing a significant increase in the density of myosin IIB puncta in the rear and front central regions of the AW with respect to the peripheral lamellipodia structures. (*n* = 10 AWs). **(G)** Histogram showing that the density of the myosin IIB puncta is significantly higher in the central region of the AW than the periphery. (*n* = 10 AWs). **(H)** Plot showing the changes in the density of myosin IIB puncta between the Rear and Front sections of the AW. **(I)** Histogram showing that the density of the myosin IIB puncta is significantly higher in the region immediately behind the AW (Proximal) than the region immediately after it (Distal). (*n* = 10 AWs). Student's test was performed for histograms: ^**^P ≤ 0.01. **(J)** Plot showing the changes in the density of myosin IIB puncta between the region immediately behind and after the AW. Increase in density in **(H,J)** is depicted in blue, decrease in red and stable (change < 12%) tendency is depicted in gray. A conceptual scheme for ROIs used for analysis is found in Supplementary Figure 5.

Next, we investigated the importance of Myosin IIB in the structure and dynamics of the AWs, and their similarities and differences with the GC ultrastructural organization. In GCs, Myosin IIB is localized mainly in the T zone, in the C domain and in the contractile node region (Rochlin et al., 1995; Loudon et al., 2006; Burnette et al., 2008). Since Myosin IIB is required for maintaining GC shape, polarization, size, actin organization, and for normal rates of shape change and traction force (Bridgman et al., 2001), we speculated that it could play a role also in AWs framework, beside the pulling effect observed herein. Recent works have highlighted that AWs generate traction forces that help their anterograde movement (Katsuno et al., 2015) suggesting the importance of Myosin II in the AW dynamics (Flynn et al., 2009). However, none of them have studied the distribution of Myosin IIB in propagating AWs in detail. To address this point, we employed two-color STimulated Emission Depletion (STED) nanoscopy (Göttfert et al., 2013) to verify whether and where Myosin IIB is present in AWs (Figure 4 and Supplementary Figure 5) with a sub-diffraction resolution. The utilization of a super-resolution technique allowed us to explore in greater detail Myosin IIB localization not only in the neurite shaft and in the propagating AWs, but also in the GC with respect to actin and β-III tubulin. This latter cytoskeletal component was acquired in confocal mode. STED images showed that Myosin IIB appeared as spots localized mostly at the sides of the β-III Tubulin (Figures 5A,B) with a net border between the central and the peripheral region of the AW (Figures 5D,E and Supplementary Figures 6A–C). The averaged line scan analysis of 16 neurites (Figure 5C) highlighted an increase in β-III Tubulin fluorescence intensity in and behind the AW that reflects an increased number of microtubules (MTs), in accordance with previous observations (Winans et al., 2016). Myosin IIB is present in the AW, with a peak at about 10 μm from the wave front that precedes by 5 μm the peak of actin, indicating a possible clustering of Myosin IIB at the rear of the advancing AWs. The quantification of the Myosin IIB puncta density revealed a significant concentration in the central region of the AWs, with a mean density of 7.3 ± 1.6 puncta/μm^2^ and a preferential localization in the rear part of the wave (8.2 ± 2.0 puncta/μm^2^; Figures 5F,G). This tendency was present in 60% of the analyzed AWs, whereas in 20% of cases the distribution appeared stable and in another 20% a slight increase toward the AW front was observed (Figure 5H). A comparison of the Myosin IIB puncta density between the regions immediately before and after the traveling AW revealed an aggregation behind the wave (5.8 ± 1.0 puncta/μm^2^ behind vs. 3.4 ± 0.8 puncta/μm^2^ in front) (Figures 5I,J).

**Figure 6:**
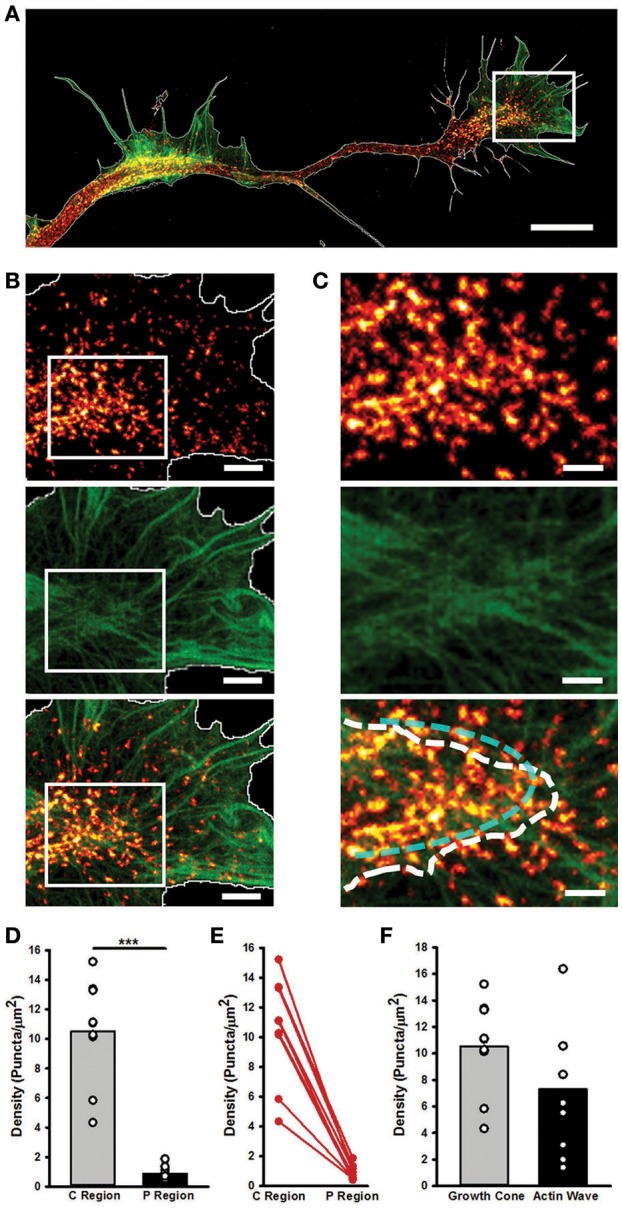
STED microscopy highlights GC and AW similarities and differences in myosin IIB localization. **(A)** Merged Image showing the Myosin IIB and actin channels of the neurite in Figure 4. The edge is highlighted in white. Scale bar = 5 μm. **(B)** Magnifications of the region delimited by the white box in **(A)** showing the actin (top panel), Myosin IIB (middle panel) and both (bottom panel) channels. Scale bar = 1 μm. **(C)** Magnifications of the area delimited by the white boxes in **(B)** of the border between the central and peripheral regions of the GC. The white dashed line defines the border between the central and peripheral regions of the GC, while the cyan dashed line highlights the acto-myosin contractile arcs of the transition zone. Scale bar = 500 nm. **(D)** Histogram showing that the density of the Myosin IIB puncta is significantly higher in the central and transition zones than in the peripheral region. (*n* = 9 GCs). **(E)** Plot showing the constant decrease (depicted in red) in the density of Myosin IIB puncta between the Central and Peripheral regions of the GC. **(F)** Histogram showing the density of Myosin IIB puncta in the GC with respect to the correspondent AWs. (*n* = 9 neurites). Student's test was performed for histograms: ^***^P < 0.001.

STED nanoscopy also allowed us to characterize the structural differences between AWs and GCs. Myosin IIB appeared as spots localized in the transition and central regions of the GC, in correspondence of dense, actin filaments (Figures 6A,B), where it showed an arc-like organization (Supplementary Figures 6D,E). Indeed, the density of the Myosin IIB spots in the central and transition regions of the GC was significantly higher (10.5 ± 1.3 puncta/μm^2^) than in the peripheral zone (0.9 ± 0.2 puncta/μm^2^), and no significant difference in the Myosin IIB concentration between the GC and the AW was found (Figures 6C–E). The analysis of linear profiles along different sections of AWs and GCs showed that in the central regions Myosin IIB puncta are isolated or cluster together along actin-rich structures, whereas in the peripheral regions isolated spots of Myosin IIB are present along thicker actin filaments. However, the arc-like, anti-parallel structure of Myosin IIB molecules and actin filaments present in the transition zone of the GC is completely lost in the AWs (Supplementary Figure 7), where the transition is sharper and flattened along the neurite shaft. Taken together, these findings indicate that, despite being similar in many aspects, the acto-myosin organization of AWs and GCs retains some crucial differences that reflect on the distinct dynamics of the two structures.

### Myosin IIB inhibition abolishes GC “pulling” effect

**Figure 7:**
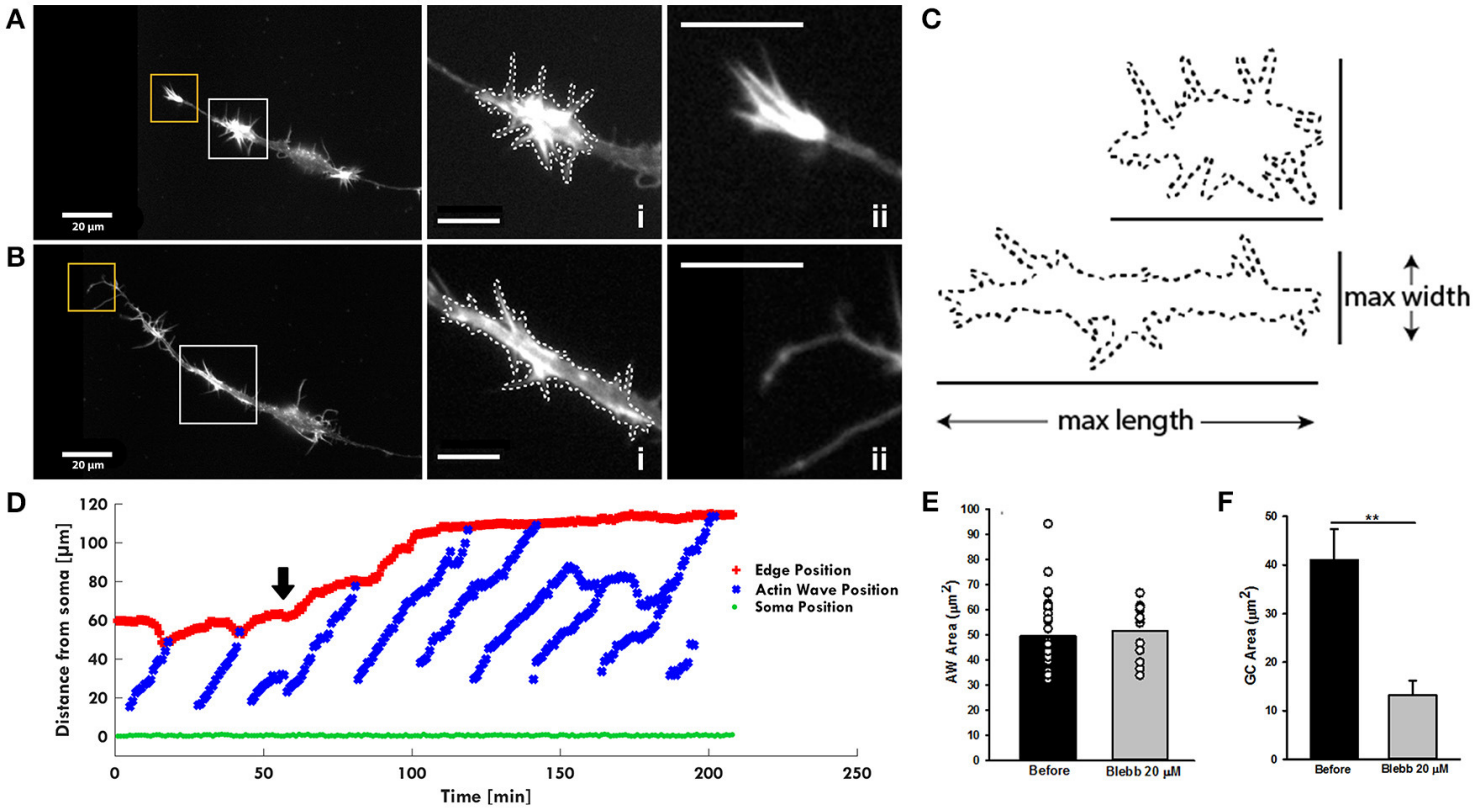
Blebbistatin treatment differently impacts AWs and GCs. **(A,B)** Live-cell images of an AW before **(A)** and after **(B)** Blebbistatin treatment (20 μM) (Supplementary Video 6). Images were collected every 10 seconds. Insets on the right represent a magnified view of the AWs (i) and GC (ii) showed in **(A,B)**. Blebbistatin treatment changes the morphology of the wave that loses the characteristic growth cone shape and abolishes the GC completely. White dashed lines indicate the AWs' shape before **(A)** and after **(B)** the treatment with Blebbistatin. Scale bar in **(A,B)** = 20 μm. **(C)** Comparison of the AWs shape before **(A)** and after **(B)** Blebbistatin treatment. **(D)** Plot highlighting the progression of the AWs (blue) shown in **(A,B)** and Supplementary Video 6 from the soma (green) along the neurite. The origin of the axes is set to the initial soma position. The black arrowhead indicates blebbistatin addition: Myosin II inhibition progressively abolishes the pulling effect. The edge trace (red) highlights the disappearance of the GC retraction upon the AW arrival after blebbistatin treatment. **(E)** Quantification of the AWs mean area (μm^2^) calculated on neurons treated with or without blebbistatin for 4 h and imaged as in **(A,B)**. *N* = 18 AWs. **(F)** Quantification of the GC mean area (μm^2^) calculated on neurons treated with or without blebbistatin for 4 h and imaged as in **(A,B)**. *N* = 6 GC. Student's and U-Man Whitney tests were performed for all histograms: ^**^P < 0.01.

After having assessed the presence of Myosin IIB in both AWs and GCs, we investigated the mechanisms regulating the pulling effect of the approaching AW on the GC, since this appears to be a basic feature of their dynamics. We speculated that the pulling effect could originate from a retrograde shift of actin likely caused by the interplay between the acto-myosin complex and the membrane of the neurite itself. To this aim, we treated hippocampal cultures with blebbistatin, which inhibits the ATP activity of the Myosin A and of B isoforms (Kovács et al., 2004). Treatment with 20 μM blebbistatin abolishes the growth cone-like shape of the AWs (Figure 7A and Supplementary Video 6) suggesting a rearrangement of the actin assembly after Myosin inhibition (Figure 7Bi). Indeed, the AWs became longer and thinner, but the area of AWs after blebbistatin addition remains almost unchanged, even if the lamellipodia structures disappear (Figures 7C,E). In contrast, the GC of all treated neurites becomes a pointed bundle of actin (Figure 7Bii), loses all lamellipodia, and its area shrinks by more than 60% (Figures 7B,F). This result suggests a higher impact of Myosin II inhibition on the structure of the GC than that of the traveling AW. Following blebbistatin treatment, the tip of the neurite exhibits a significant outgrowth and the progressive disappearance of the pulling effect when the AW approaches the tip of the neurite. This could be the effect of the inability of the GC to move (Figure 7D). We then determined the frequency of the AWs calculating the number of complete waves (i.e., waves able to reach the tip of the growth cone) in an hour. Blebbistatin increased the frequency of AW appearance and, at the same time, reduced AWs velocity, calculated as the time taken by the wave to reach the tip of the growth cone, normalized for the length of the neurite (Flynn et al., 2009; Katsuno et al., 2015; Tilve et al., 2015) (Supplementary Figure 8). Overall, these data show a strong involvement of Myosin II in both structural and dynamical organization of AWs, and highlight a dissimilarity in the role of Myosin in AWs and in the GC itself. In both GCs and AWs Myosin seems to be responsible for the maintenance of lamellipodia.

While in the GC myosin inhibition causes a rapid neurite outgrowth, the AWs traveling speed is reduced and the waves loses their GC-like shape. Since Myosin is important for the retrograde actin flow in both structures—as found in the recent work by Katsuno et al. (Katsuno et al., 2015)—we can assume that the simultaneous disruption of shape and retrograde flow in both structures upon blebbistatin inhibition makes impossible to exert the forces needed to mediate the “pulling” effect and finely regulates the neurite outgrowth.

### Cholesterol depletion impacts AW formation and morphology

**Figure 8:**
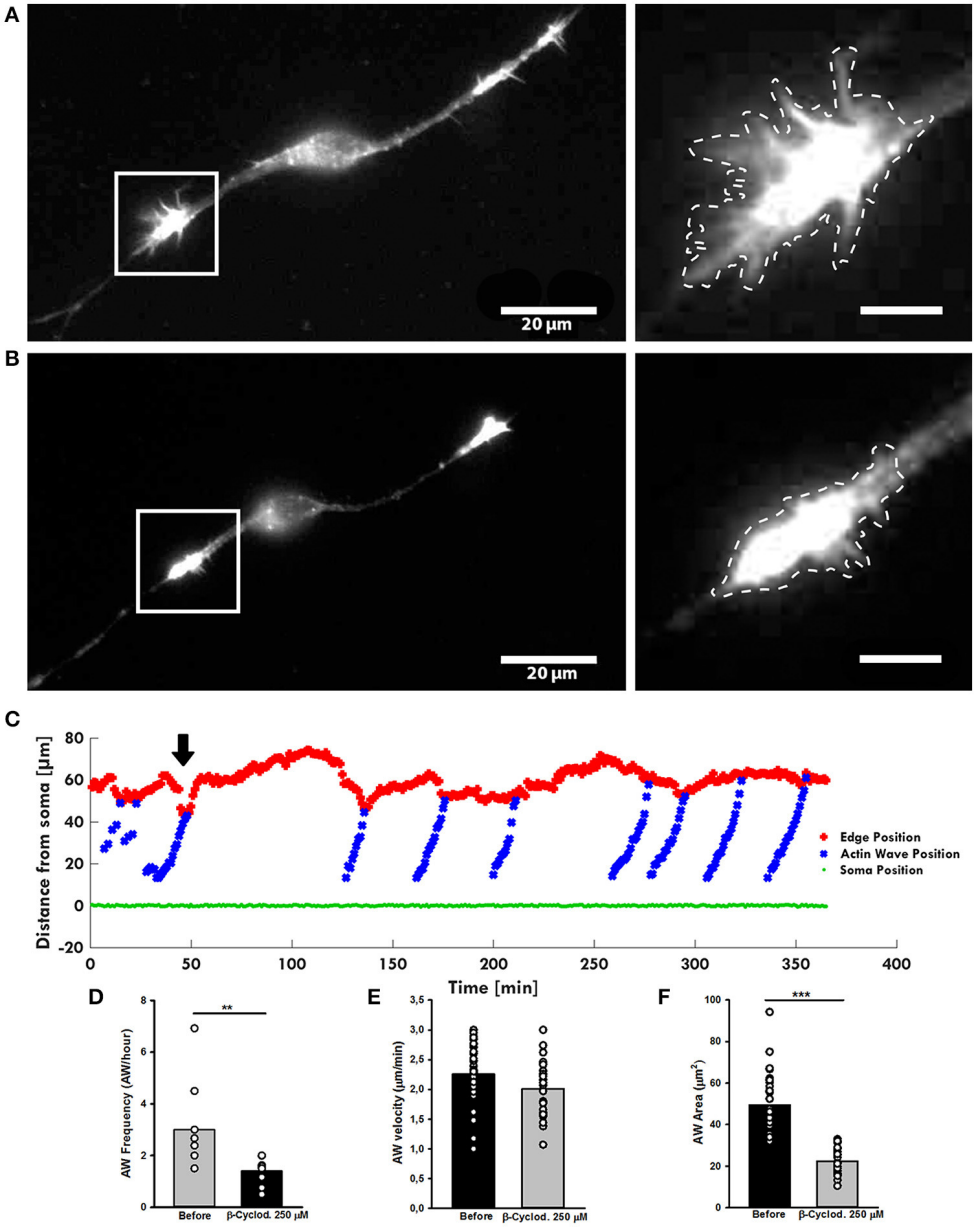
β-cyclodextrin treatment affects AWs structure. **(A,B)** Live-cell images of an AW before **(A)** and after **(B)** β-cyclodextrin treatment (250 μM). On the right, magnified views of the AWs showed in **(A,B)** before and after β-cyclodextrin treatment that abolishes completely the growth cone-like shape. Images were collected every 10 s. Dashed lines indicate the AW's shape before **(A)** and after **(B)** the β-cyclodextrin treatment. Scale bar in **(A)** = 20 μm. Scale bar in **(B)** = 5 μm **(C)**. Plot highlighting the progression of the AWs (blue) showed in **(A,B)** from the soma (green) along the neurite. The origin of the axes is set at the initial soma position. The black arrowhead indicates β-cyclodextrin addition: cholesterol depletion decreases the neurite retraction. The edge trace (red) highlights the slight reduction of the GC retraction upon AW arrival after β-cyclodextrin treatment **(D)** Quantification of the AWs frequency calculated on neurons before and after β-cyclodextrin treatment for 4 h and imaged as in **(A,B)**. *N* = 9 neurites. **(E)** Quantification of the AWs velocity (μm/min) calculated on neurons treated with or without β-cyclodextrin for 4 h and imaged as in **(A,B)**. *N* = 16 AWs. **(F)** Quantification of the AWs mean area (μm^2^) calculated on neurons treated with or without β-cyclodextrin for 4 h and imaged as in **(A,B)**. *N* = 16 AWs. Student's and U-Man Whitney tests were performed for histograms: ^**^P < 0.01; ^***^P < 0.001.

As inhibition of Myosin II alters the shape of AW, the surface tension of the membrane surrounding the wave could have a role in its dynamics. A high cholesterol concentration increases the membrane tension and its reduction has been demonstrated to reduce the stiffness of the cellular membrane enveloping the actin filament network (Amin et al., 2012). To test the effect of the cholesterol depletion on the AWs dynamic, we treated the hippocampal neurons with the β-cyclodextrin that has been reported to be the most efficient compound in extracting cholesterol from membranes (Vladislav et al., 2013). Live-cell imaging showed a loss of the growth cone-like shape of the AW after addiction of β-cyclodextrin (Figures 8A,B), a reduction of the AW's pulling effect (Figure 8C), and a severe loss of the AWs' area (Figure 8F). Treatment with β-cyclodextrin reduced both velocity and frequency of AWs by about 10 and 50% respectively (Figures 8D,E). No visible effect was observed on the GC after treatment with β-cyclodextrin. Taken as a whole, our data suggest that the membrane is involved not only in AW shape maintenance, but also in AW dynamics.

### RhoGTPases regulate AW architecture and dynamics

RhoGTPases modulate actin nucleation and play a key role in the regulation of GC morphology and dynamics (Dickson, 2001). They are known to act downstream of a plethora of signaling molecules and guidance cues, and to influence the activity of actin and actin-binding molecules (Hall and Nobes, 2000; Lowery and Van Vactor, 2009; Toriyama et al., 2013). Hence, we wondered whether they have a similar role also in AWs.

**Figure 9:**
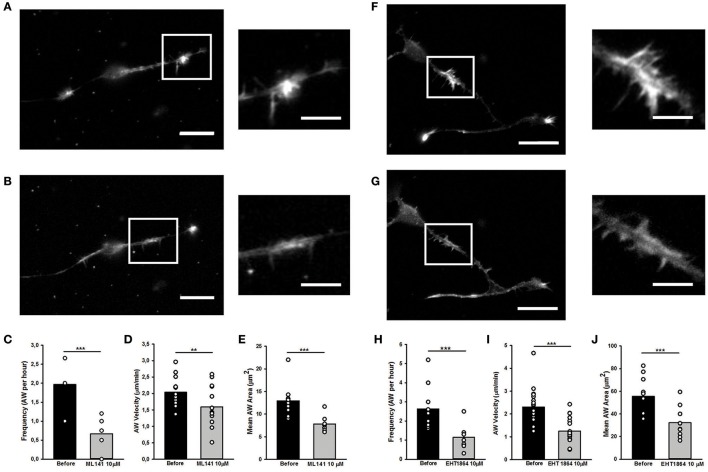
Rho GTPases inhibition affects AW morphology and dynamics **(A,B,F,G)** Live cell imaging of DIV2 rat hippocampal neurons before **(A)** and after **(B)** addition of ML141 (10 μM) and before **(F)** and after **(G)** addition of EHT1864 (10 μM). White boxes indicate the growth cone-like AW. On the right, magnified views of the AW shown in **(A,F)** and **(B,G)** before and after drugs treatment. Scale bar in **(A,B)** = 20 μm. Scale bar in **(F,G)** = 10 μm. **(C,H)** Quantification of the AWs frequency calculated on neurons treated with or without ML141 **(C)** and with or without EHT1864 **(H)** for 4 h and imaged like in **(A,F)** and **(B,G)**. (*n* = 10 neurites). **(D,I)** Quantification of the AWs velocity (μm/min) calculated on neurons treated with or without ML141 **(D)** and with or without EHT1864 **(I)** for 4 h and imaged like in **(A,F)** and **(B,G)**. (*n* = 10 AWs for ML141 and *n* = 10 AWs for EHT1864). **(E,J)** Quantification of the AWs mean area (μm^2^) calculated on neurons treated with or without ML141 **(E)** and with or without EHT1864 **(J)** and imaged like in **(A,F)** and **(B,G)**. (*n* = 9AWs for ML141 and *n* = 10AWs for EHT1864). Student's test was performed for all histograms: ^**^P < 0.01;^***^P < 0.001.

Since Rac1 and Cdc42 are known to play a part in anterograde propagation of an AW (Winans et al., 2016), we started our study by assessing the function of these NPFs. Cdc42 and Rac1 can be inhibited by the selective guanine nucleotide binders ML141 and EHT1864, respectively, providing a reversible and non-competitive inhibition (Shutes et al., 2007; Surviladze et al., 2010). Cdc42 inhibition (ML141, 10 μM) altered the GC shape (Figures 9A,B), decreased AWs velocity (−25%), size (−40%), and frequency 60 min after drug addition (−75%) (Figures 9C–E). These effects are concentration-dependent, since ML141 at 30 μM abolishes the wave phenomena without a significant change in the GC dynamics (Supplementary Figure 9).

Similarly to the Cdc42 inhibition, the addition of Rac1 inhibition by EHT1864 at 10 μM concentration caused a complete loss of the growth cone-like shape (Figures 9F,G) and a significant reduction of the AW Area (Figure 9J). Moreover, a reduction of 75% in AWs frequency (Figure 8H) together with a 50% decrease in the AWs velocity (Figure 9I) assessed an important role for Rac1 in positively modulating the actin polymerization required for the AWs generation. These findings highlight the role of Rac1 in wave inception and are consistent with previous data, where its activation was sufficient to trigger AW initiation and protrusion (Winans et al., 2016).

Taken together, these data support the hypothesis that Cdc42 and Rac1 have an important role in the AW architecture and dynamics, and suggest a strongest role of Cdc42 in AW inception and of Rac1 in AW traveling, hence pointing at the presence of different actin-regulating mechanisms involved in different steps of the AW biology.

## Discussion

The function and role of AWs in neuronal development is still unclear. In the present work, we show that migrating AWs do not boost neurite outgrowth and we suggest an alternative role for it. While the GC size is reduced in the absence of AWs, the arrival of AWs solely restores the original GC extension allowing it to properly explore the environment (Figure 4). We also demonstrate that even if AWs resemble the GC in size and appearance, there are important differences in the acto-myosin complex that in the AWs is more involved in shape maintenance, while in the GC it controls both structure and dynamic. In conclusion, we provide clear evidence for the involvement of both membrane and small GTPases in the regulation of the speed and shape of migrating AWs. Let us now discuss in detail the possible functions of AWs and the underlying molecular mechanisms.

### Functions of AWs

The AWs have been originally described in 1998 by Ruthel and Banker as wave-like membrane protrusions containing actin filaments along the axons and immature neurites of cultured rat hippocampal neurons. These authors also highlighted a key role for the AWs in transporting actin and associated proteins to the GC at the tip of an extending axon (Ruthel and Banker, 1998). Following their work, different groups have then found a dual role for AWs in neurite outgrowth *in vivo*: they are the drivers in neurite extension and, at the same time, they play an important role in a stochastic search mechanism that allows a set of neurites to explore the surrounding space to sense the polarized growth cues provided by the developing brain (Flynn et al., 2009; Katsuno et al., 2015; Winans et al., 2016). AWs investigated herein are generated at a frequency of 2–3 waves per hour, propagate with an average speed of ~2–3 μm/min and travel from the base of the neurites to the GC. Our long-term live cell imaging experiments clearly show that the continuous arrival of AWs generates retraction/growth cycles that, although causing a transient increase of the GC size, do not result in a net neurite elongation (Figures 1–3). A possible explanation for the discrepancy between our results and what previously reported could be related to the use of P1–P2 hippocampal neurons in our experiments, whereas previous studies used embryonic mouse (E16–E17) or rat (E18) (Flynn et al., 2009; Katsuno et al., 2015; Winans et al., 2016; Tomba et al., 2017). Indeed, although neurogenesis continues postnatally, many more neurons are born at the earlier stages and the “older” neurons might exhibit a different behavior. It is also worth pointing out that our manuscript is the first one to give a complete picture of the long-term behavior of the AWs that, despite not promoting neurite outgrowth, seem to mostly contribute to the stochastic “tug of war”-like growth and retraction that leads to neuron polarization, as already suggested in other papers (Flynn et al., 2009; Winans et al., 2016; Inagaki and Katsuno, 2017). Another possible role of the AWs is suggested by the experiments similar to those shown in Figure 1 and summarized in Figure 4: during the exploration of the environment, GCs become smaller and following the arrival of an AW, GCs recover their original area suggesting that AWs have certainly the role and function of providing fresh actin oligomers and other metabolic products which were lost during the exploration of the environment. Therefore, we propose that AWs have a major role in maintaining the GC lively and active, so that filopodia can remain sufficiently long to explore the environment and lamellipodia can maintain their structure and architecture (Figure 4B).

### Morphology and architecture of AWs and GCs: similarities and differences

AWs resemble the GC in size and appearance: both structures bear filopodia and lamellipodia (Ruthel and Banker, 1998; Lowery and Van Vactor, 2009) and are enriched with actin and actin-associated proteins (Ruthel and Banker, 1998; Flynn et al., 2009; Toriyama et al., 2013; Kubo et al., 2015) that modulate their structure and movement. Actin and molecules that modulate its dynamics are present in both structures, and contribute to the balance between treadmilling and retrograde flow that produces the traction force on the substrate (Toriyama et al., 2013; Katsuno et al., 2015; Inagaki and Katsuno, 2017). STED nanoscopy revealed the AWs structure with a sub-diffraction resolution: similarly to what observed in the GC, in the AWs F-actin is present in filopodia (bundled F-actin) and lamellipodia (F-actin meshwork). The distribution of Myosin IIB puncta appear to be also similar: it accumulates in the central regions of both GC and AWs, especially in the regions where actin filaments are more densely condensed (Figure 5). However, the acto-myosin contractile arcs, that in the GC support the retrograde flow and divide the microtubule-rich central domain from the peripheral region, are substituted in the AW with linear, dense acto-myosin structures organized in a rear-to-front gradient, that separate the main core of the AW from the erupting peripheral elements (Supplementary Figures 6, 7). This difference can be explained with the diverse motion patterns of GCs and AWs: while the former needs to expand and retract to better explore the surrounding environment for neurite elongation (Dent et al., 2011; Coles and Bradke, 2015), the latter travels along the neurite shaft and needs stable, bilateral structures that can propel it forward modulating the acto-myosin dynamics and the forces exerted to the substrate, in concert with upstream signaling molecules (Toriyama et al., 2013; Katsuno et al., 2015). Furthermore, in agreement with previous findings, our results indicate that Myosin IIB has a role that goes far beyond the architecture maintenance: indeed, as an AW approaches the distal neurite, the GC retracts by several microns so to merge with the incoming AW and then advances again (Figure 1). After treatment with blebbistatin, the retrograde motion of the GCs was abolished together with a significant neurite outgrowth (Figure 7D) confirming quantitative measurements done by Flynn et al. (2009). These findings strongly suggest that Myosin IIB activity normally regulates the rate of retrograde flow that pulls the neurite backwards (Katsuno et al., 2015): its absence triggers a strong increase in actin-bundle length. This observation is in agreement with the blebbistatin experiments performed by Flynn and co-workers, who found a decrease in the GC retraction upon low concentration of blebbistatin treatment (Flynn et al., 2009), and supports the view that the “push” of actin assembly and the “pull” of myosin, that disrupts the actin filaments at their rear, combine to guide the retrograde flow (Medeiros et al., 2006). In recent studies, such acto-myosin structural and dynamic interaction was sufficient to induce contractile t-waves in *in vitro* models (Reymann et al., 2012). If the shape and morphology of AWs and of GCs are similar, these two structures present a very different drug response: blebbistatin treatment leads to a substantial collapse of the GC but not of the AW (Figures 7A,B). This difference in sensitivity can be rationalized by taking into account a redistribution of the actin filaments packed into the wave which continues traveling along the microtubules-rich neurite shaft, driven by the constant treadmilling of actin filaments that, in concert with adhesion proteins like shootin-1, might be capable of exerting traction forces to drive AWs forward (Katsuno et al., 2015). On the other hand, the GC structure is finely regulated by acto-Myosin arcs in the transition zone that, besides regulating the retrograde flow (Van Goor et al., 2012), keep the microtubules packed in the central region. The prominent role of Myosin IIB in regulating these dynamics affects the GC architecture.

### Mechanisms of AWs migration: membrane and membrane-bound regulatory proteins

Actin is known to be associated with membranes and there have been a number of theoretical studies that focus on the connection between the protrusive forces generated by actin polymerization and the dynamics of the membrane-bound activators. Indeed, F-actin network assembly, organization and dynamics are controlled by the spatial and temporal regulation of the activity of actin-binding proteins that are associated with the membrane in a multifaceted way (Bezanilla et al., 2015). To explore the influence of the membrane on the AWs' dynamic, we treated our DIV2 hippocampal cells with β-cyclodextrin, responsible for the depletion of cholesterol from the membrane (Amin et al., 2012). In line with the role of the membrane in the AWs' structure, the elimination of cholesterol decreases AWs' area and causes a loss of the growth cone shape. Interestingly, β-cyclodextrin affects not only the morphology, but also the dynamics of wave phenomena: both frequency and velocity of AWs decreased after β-cyclodextrin addition, suggesting a role for the membrane that goes beyond AW shape maintenance. Our data suggest that the membrane may be important for the correct localization of actin filaments and actin-associated proteins in the propagation of AWs, and any perturbation that affects the dynamics of the signaling pathways involved in F-Actin polymerization/depolymerization. The same morphological effect was observed when selective inhibitors of the RhoGTPases Rac1 and Cdc42 were added to the cellular medium. Indeed, the Rho-family GTPases represent a key node for connecting extracellular signals to regulated actin dynamics and, by stimulating actin dynamics, they induce plasma membrane protrusion such as lamellipodia and filopodia (Hall and Nobes, 2000; Burridge and Wennerberg, 2004). Recently, Winans and co-authors demonstrated that Cdc42 exhibit a higher activity in front of an AW, whereas Rac1 activity increases more broadly in the whole AW, and that its activation is sufficient to initiate AWs eruption and propagation (Winans et al., 2016). Indeed, we found that the frequency and the velocity of the AWs, used as parameters of neurite dynamics, decreased in the presence of both the RhoGTPases inhibitors. This significant decrease in AWs initiation capacity and propagation kinetics can be explained with a putative role of these two RhoGTPases in the regulation of both actin polymerization and assembly through the Arp2/3 complex or formins and adhesions strength through an increase in shootin-1 phosphorylation that has been shown to mediate the linkage between F-actin retrograde flow and cell adhesion in GCs (Kubo et al., 2015). A cascade downstream of Cdc42, Rac, and subsequently PAK1 can be considered responsible for the modulation of the balance between adhesion strength and Myosin mediated retrograde flow in maintaining anterograde stable unidirectional motion. Moreover, we observed that Cdc42 inhibition induces a burst in the neurite outgrowth suggesting that it could contribute to the acto-myosin contractility required for modulation of the neurite outgrowth (Wilkinson et al., 2005). Taken together, these results seem to indicate that Cdc42 and Rac1 could promote AWs migration toward the leading edge and could be involved in the assembly and disassembly of F-actin network also in the AWs. However, the precise spatio-temporal modulation of these RhoGTPases, and of RhoA, that can be an important player for its myosin modulation dynamics, is still unclear, and further experiments to correlate the reciprocal dynamics of these players are needed to fully understand their interactions in the modulation of AWs' inception and movement.

In conclusion, in our study we have analyzed the dynamics of AWs and AW constituent proteins to unravel the mechanisms of wave inception and propagation. Using live cell imaging and STED, we have characterized their growth cone-like morphology and motility and we have provided evidence that Myosin II, membrane composition, as well as small RhoGTPases act as critical components of AW dynamics. Our study suggests the existence of a cross-talk between actin cytoskeleton and its upstream regulators such as RhoGTPases, the motor protein Myosin and the cellular membrane integrity. These interactions are necessary to translocate the actin and associated proteins along the neurite.

## Author contributions

VT and LN designed the research; SM and FI performed live-cell imaging experiments; ED performed STED experiments; AP developed the software for the analysis of live-cell imaging experiments; SM, FI, LN and VT analyzed data from live-cell imaging experiments; ED and DC analyzed data from STED experiments; VT, LN, SM and FI wrote the original draft; all the authors contributed to the review and the editing of the manuscript; VT supervised the entire work.

### Conflict of interest statement

The authors declare that the research was conducted in the absence of any commercial or financial relationships that could be construed as a potential conflict of interest.
