# Supplementary material for: Actin Waves Do Not Boost Neurite Outgrowth in the Early Stages of Neuron Maturation
Source: Front Cell Neurosci. 2017 Dec 18;11:402. doi: 10.3389/fncel.2017.00402 (PMC5741660; doi:10.3389/fncel.2017.00402)
Supplement: Supplementary file 7 [file DataSheet1.pdf]

Supplementary Material

Actin Waves do not boost neurite outgrowth in the early stages of neuron maturation.

S. Mortal[1,*], F.Iseppon[1,*], A. Perissinotto[1], E. D'Este[2], D. Cojoc[3], L.M.R. Napolitano[1], and V. Torre[1]

[1] International School for Advanced Studies, Trieste 34126, Italy;
[2] Department of NanoBiophotonics, Max Planck Institute for Biophysical Chemistry, 37077 Göttingen, Germany
[3] Optical Manipulation Lab, CNR-IOM, Trieste 34149, Italy;

[*]These authors equally contributed to this work

To whom correspondence should be addressed: Vincent Torre; e-mail: torre@sissa.it.

Keywords: Actin Waves (AWs); Growth Cones (GCs); Myosin IIB; ß-cyclodextrin; RhoGTPases.

1.1 Supplementary Figures

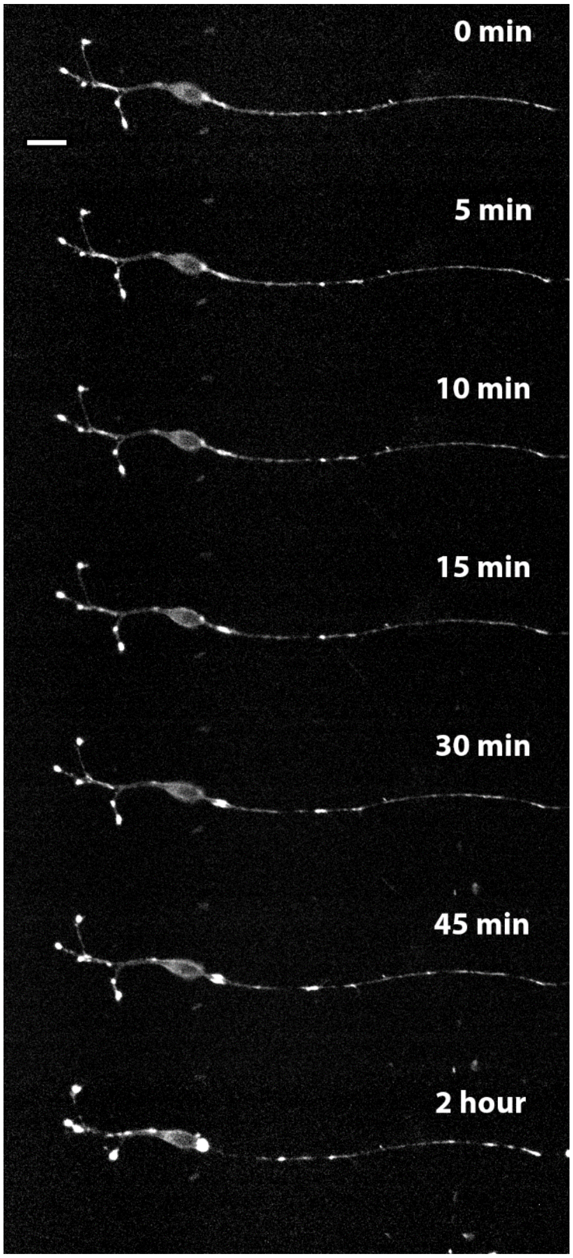

Supplementary Figure 1. SiR-Actin induces a decrease of AWs. Timelapse images of a hippocampal cell expressing the membrane-permeable SiR-Actin [1 μM]. We observed the absence of AWs presumably due to the presence of jasplakinolide (Bubb et al., 1994; Winans et al., 2016). Scalebar = 20 μm.

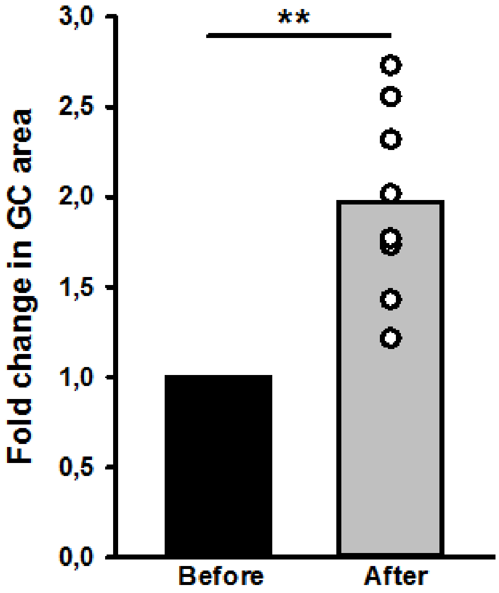

Supplementary Figure 2. Wave arrival affects GC morphology. Histogram showing a significant increase in GC area after the arrival and merge of an AW (n=8 AWs). The data were normalized by the GC area computed before the AW arrival. Student's test was performed for histograms: **P<0.01.

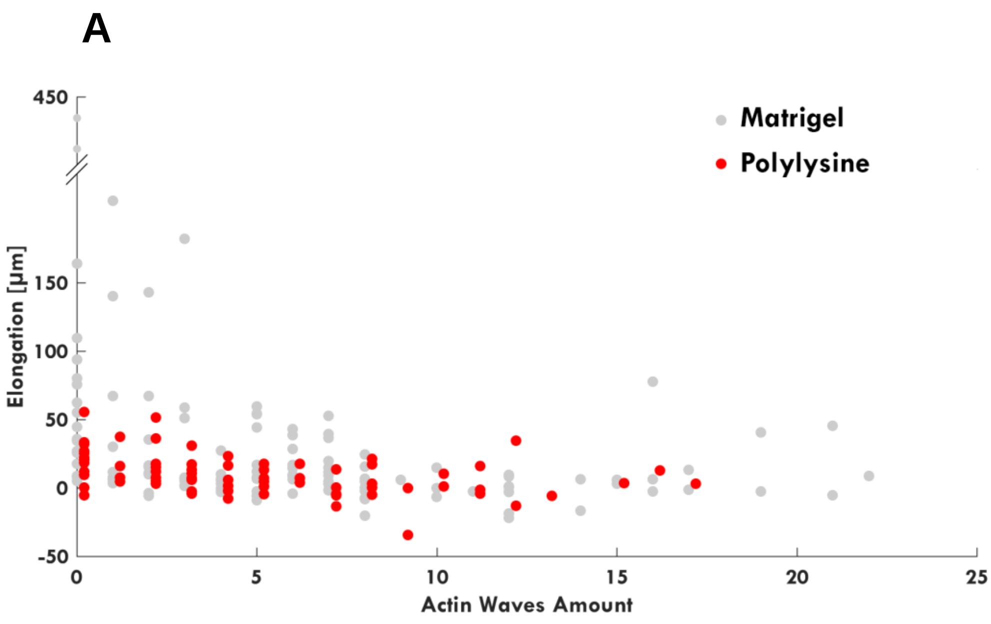

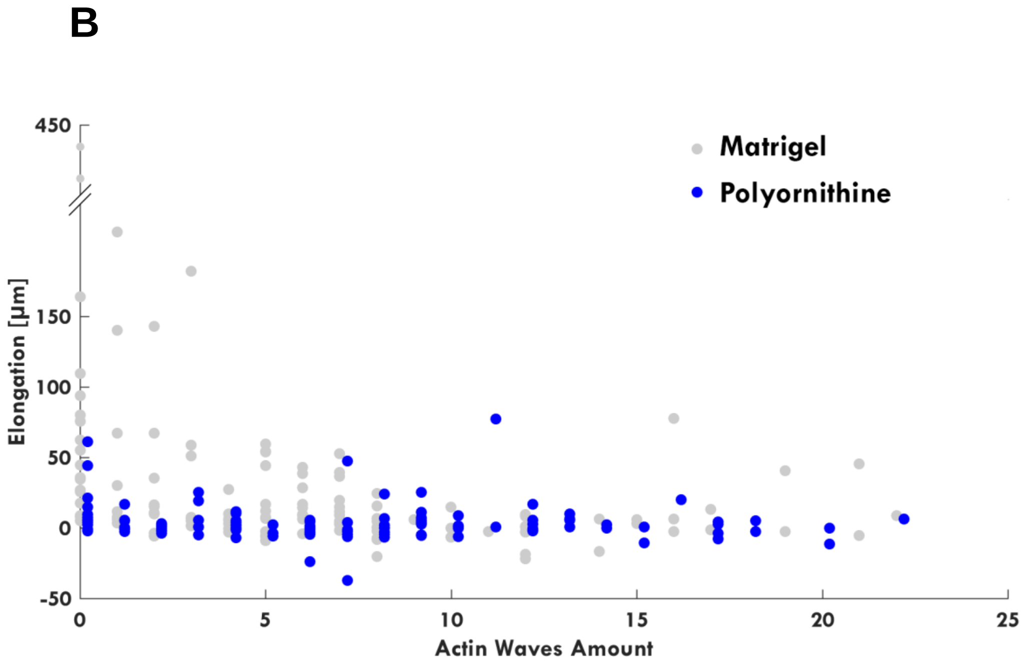

Supplementary Figure 3. AWs behaviour on different substrates. (A-B) Graph showing the elongation of the neurites (y axis) per number of actin waves (x axis) imaged at 1-5 minutes intervals for 8 hours in the presence of different substrates. In all three cases we were able to observe that AWs do not boost neurite outgrowth and neurites without AWs can elongate more. According to the literature (Dent et al., 2007), neurites could grow more on Matrigel than on the other substrates. Red dots refer poly-D-lysine n=76; light grey dots refer to Matrigel; blue dots refer to poly-L-ornithine n=122.

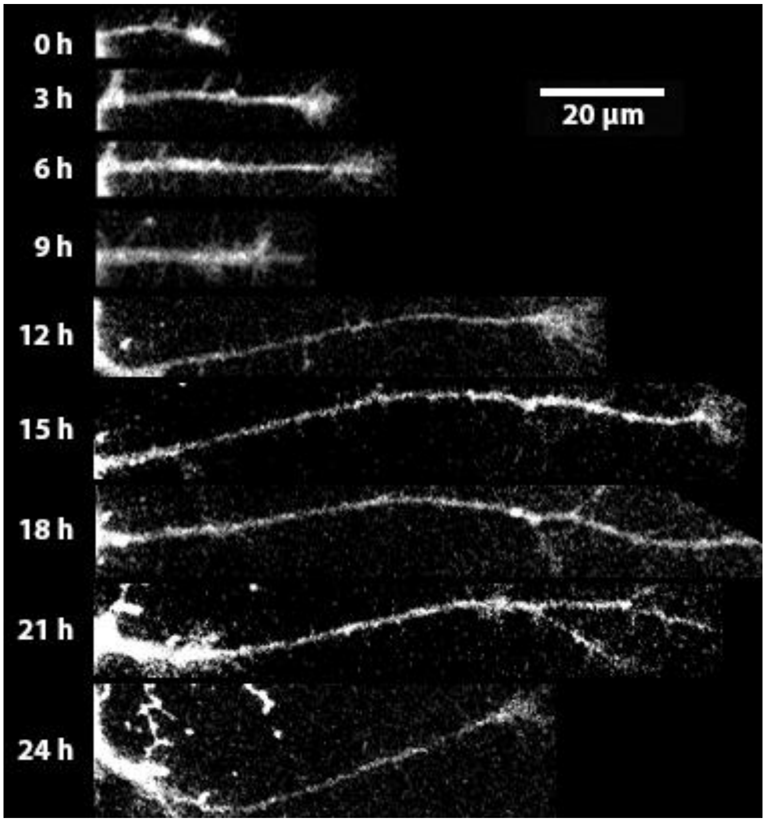

Supplementary Figure 4. AWs do not induce neurite outgrowth. Timelapse images a neurite stained with Vybrant DiI and followed for 24 Hours. As highlighted in the plots showed in Figure 3C-D the neurite elongates in the absence of AWs, while it retracts in the presence of AWs. Scale bar = 20 µm

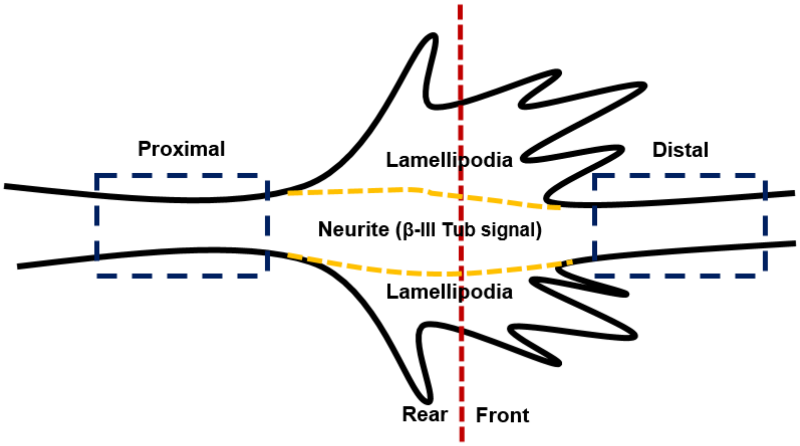

Supplementary Figure 5. STED images analysis scheme. Scheme of the concept and Regions Of Interest (ROIs) taken into consideration in the analysis of Myosin-IIB puncta. The Neurite region (yellow dashed lines) takes into consideration the central region of the AW, the prosecution of the β-III Tubulin-rich neurite, and is divided into Rear and Front (red dashed line) halves. The Lamellipodia region comprises all the peripheral structures that protrude from the central neurite. The Proximal and Distal sections (Blue dashed boxes) refer to the regions (of the same length) immediately before the beginning and after the front of the AW, respectively.

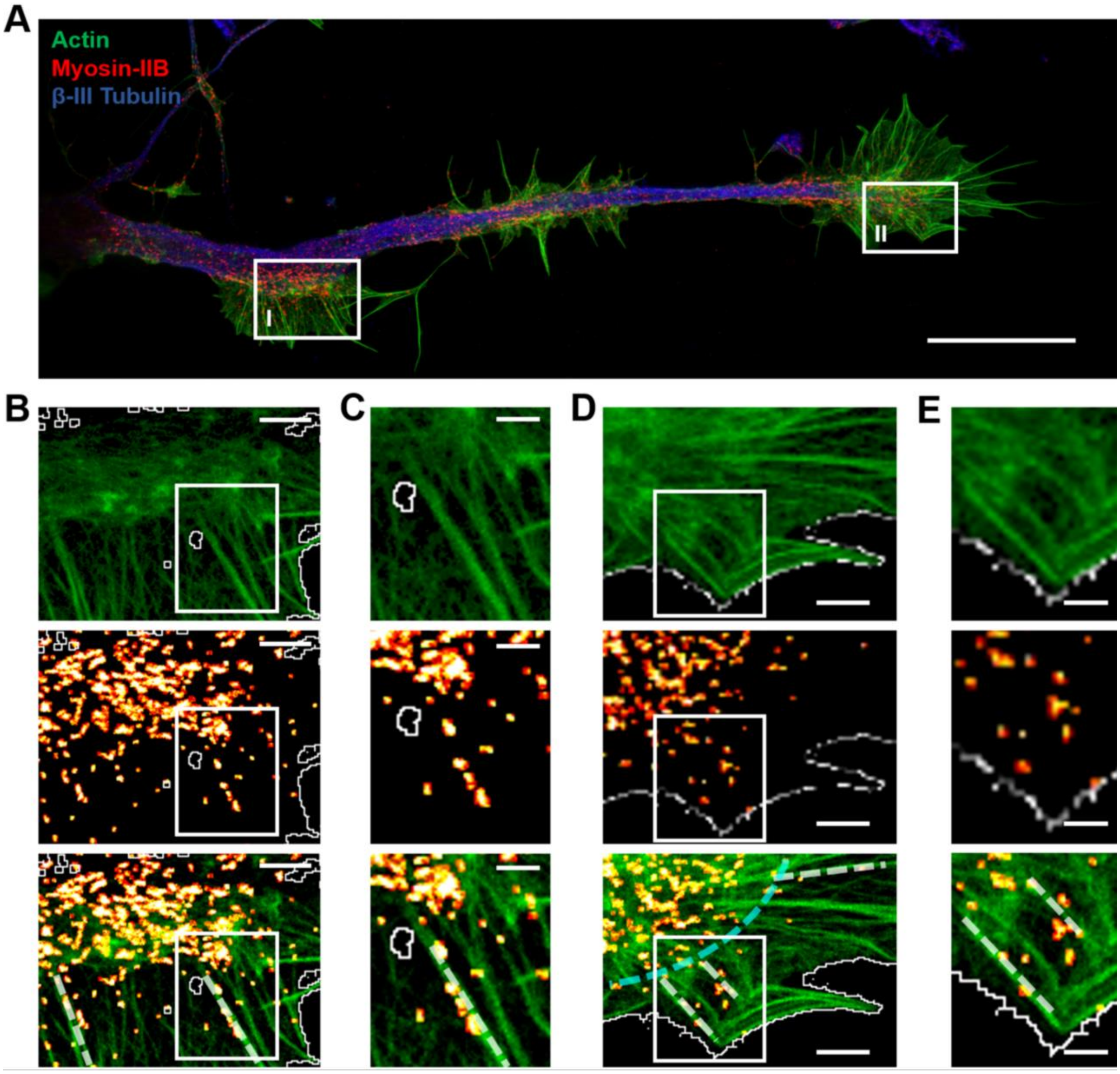

Supplementary Figure 6. Myosin central and peripheral puncta localization in GCs and AWs. (A) STED image of a rat hippocampal neuron at 1 DIV stained with actin (phalloidin, green), non-muscle myosin IIB (red) and ß-III tubulin (blue). Scalebar = 10µm **(B)** Magnifications of the region, delimited by white box "I" of the image in (A) showing the actin (top panel), myosin IIB (middle panel) and both (bottom panel) channels highlighting how myosin IIB spots change organization from a sparse localization in the central region of the AW to pinpointing along thicker actin filaments. Scalebar = 1µm **(C)** Magnifications of the area delimited by the white boxes in (B) of the border between the central and peripheral regions of the AW, with a dense actin network sparse myosin spots, and the peripheral region, where the spots organize along actin filaments, as highlighted by the white dashed lines in the bottom panels in (B, C). Scalebar = 500nm. 10µm **(D)** Same as in (B), but the magnifications refer to white box "II". In this case the magnification highlights a clear arc-like myosin organization (cyan dashed line) in the transition region between the dense central region and the periphery, where myosin puncta tend to localize along actin filaments (white dashed lines). Scalebar = 1µm **(E)** Same as in (C), but the magnified area is delimited by the white boxes in (D). Scalebar = 500nm.

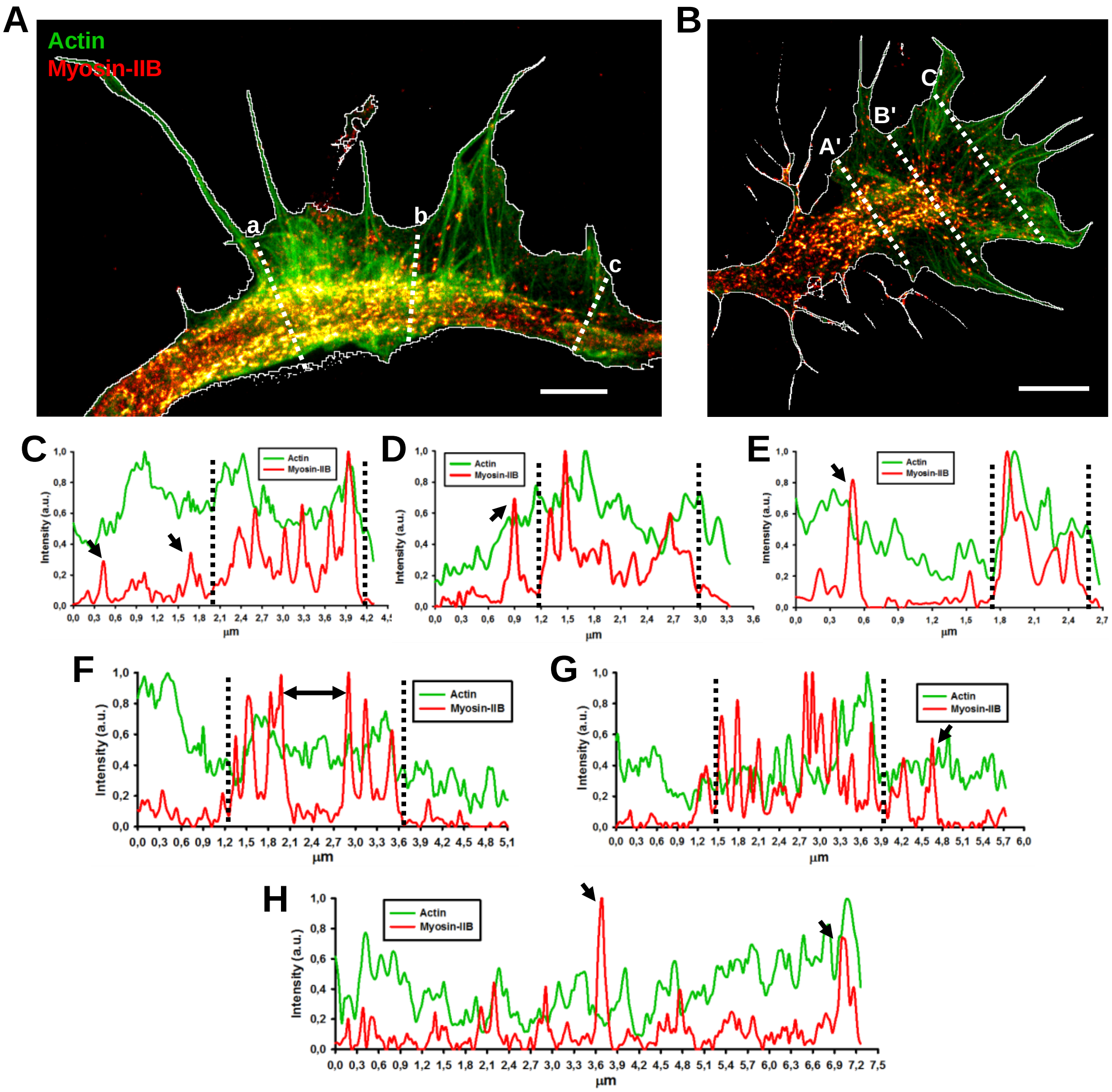

Supplementary Figure 7. Image Profiles reveal differences in acto-myosin organization between GCs and AWs. (A,B) STED images of actin (green) and myosin IIB (red) channels of the AW and GC of the cell in Figure 4. Scalebars in (A, B) = 2µm **(C, D, E)** Image profiles of actin and myosin IIB along the pointed lines in (A), showing different profiles for the rear (a→(C)), the centre (b→(D)) and the front (c→E)) of the travelling AW. The neurite central region is clearly visible in all three profiles (between the black pointed lines in (C, D, E)), where myosin IIB intensity is higher and the puncta are wider, whereas in the peripheral region single spots prevail (black arrows). **(F, G, H)** Image profiles of actin and myosin IIB along the pointed lines in (B), showing different profiles for the rear (A'→(F)), the centre (B'→(G)) and the front (C'→H)) of the GC. The central region of the GC is clearly visible in (F) and (G) (between the black pointed lines), where myosin IIB intensity is higher and the puncta are more frequent. The black double arrow in (F) highlights the lack of myosin puncta that are concentrated along the acto-myosin arcs in the transition zone. In the peripheral region (H) myosin is organized in single puncta that concentrate along actin structures (black arrows).

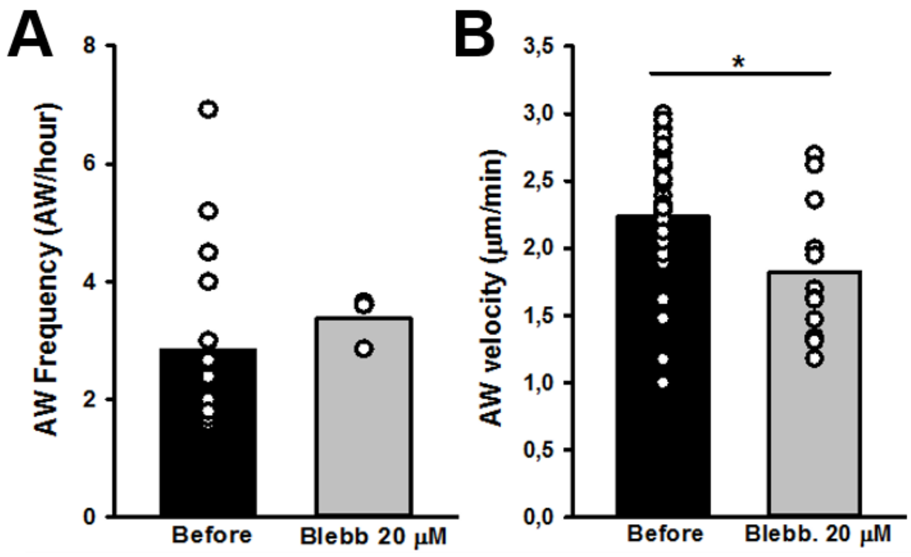

Supplementary Figure 8. Blebbistatin effect on AWs' velocity and frequency. A) Quantification of the AWs frequency calculated on neurons treated with or without blebbistatin for 4 hours and imaged like in Figure 4A-4B. N = 4 neurites **(B)** Quantification of the AWs velocity (µm/min) calculated on neurons treated with or without blebbistatin for 4 hours and imaged like in Figure 6A-6B. N = 18 AWs.

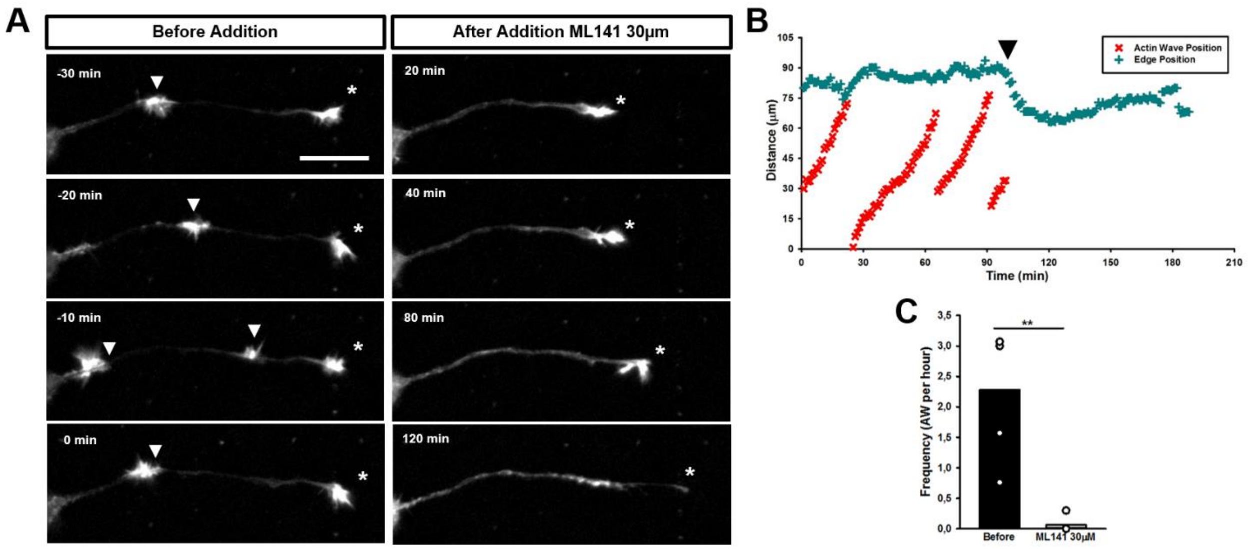

Supplementary Figure 9. Cdc42 potent inhibition affects AW dynamics. (A) Time-lapse live cell imaging of DIV2 rat hippocampal neurons before (left) and after (right) addition of ML141 (30µM). White arrow indicates the growth cone-like AW. The asterisk indicates the GC. Scale bars = 20µm. **(B)** Plot showing the progression of several AWs (red) along the neurite shaft. The edge trace (light blue) highlights neurite retraction concurrent with complete AW disappearence after ML141 treatment. Black arrowhead indicates ML141 addition. **(C)** Quantification of the AWs frequency calculated on neurons treated with or without ML141 for 4 hours and imaged like in (A) and (B). N= 9AWs. Student's test was performed for histograms: **P<0.01.

Supplementary Video

Supplementary Video 1. AWs exhibit a growth cone-like morphology and travel in an anterograde fashion in our 1 and 2 DIV imaging windows. This movie shows time-lapse images from the entire frame of acquisition (8 hrs) for mCherry-LifeAct expressing neurons. Images were collected every 5 seconds and the movie was generated at 15 frames per 1 second. Scale bar= 20 µm.

Supplementary Video 2: This movie shows time-lapse images from the entire frame of acquisition for mCherry-LifeAct expressing neurons plated on poly-D-Lysine substrate. Images were collected every 10 seconds and the movie was generated at 7 frame per 1 second. Scale bar= 20 µm.

Supplementary Video 3: This movie shows time-lapse images from the entire frame of acquisition for mCherry-LifeAct expressing neurons plated on poly-L-L-Ornithine substrate. Images were collected every 10 seconds and the movie was generated at 15 frame per 1 second. Scale bar= 20 µm

Supplementary Video 4. AWs are observed in DIV1 hippocampal neurons stained with a membrane marker. This movie shows timelapse images of a DiI-expressing neuron producing AWs. Images were collected every 5 min and the movie was generated at 29 frames per second. Scale bar= 100 µm.

Supplementary Video 5. Neurites with no or few AWs grow up to 450 µm. This movie shows time-lapse images from the entire frame of acquisition (8 hrs) for mCherry-LifeAct expressing neurons.

Images were collected every 1 min and the movie was generated at 25 frames per second. Scale bar=100 µm.

Supplementary Video 6: Blebbistatin treatment (20µM) changes the morphology of the wave that loses the characteristic growth-cone shape and abolishes completely the GC. This movie shows time-lapse images from the entire frame of acquisition (3 hrs) for mCherry-LifeAct expressing neurons. Images were collected every 10 seconds and the movie was generated at 15 frames per 1 second. Scale bar= 20 µm.

.
